# Study on the Impact of Local Heating Combined with Deflection Agitation on the Conversion Efficiency of Gel Waxy Crude Oil in Storage Tanks

**DOI:** 10.1021/acsomega.6c06251

**Published:** 2026-09-07

**Authors:** Hang Dong, Xiaowan Liang, Jian Zhao, Zhihua Wang

**Affiliations:** Key Laboratory for Enhance Oil and Gas Recovery of the Ministry of Education, 117792 Northeast Petroleum University, Daqing, Heilongjiang 163318, China

## Abstract

To address the issues of extensive low-temperature dead zones and insufficient gelled-oil conversion efficiency associated with traditional heating methods in waxy crude oil storage tanks, this study proposes an improved scheme coupling localized concentrated heating with mechanical agitation at a specific deflection angle (45°). Distinct from previous passive heat transfer approaches that solely focus on improving the geometric structure of heating elements, this work reveals the mechanism of “spatial synergistic matching between flow paths and localized fixed heat sources.” By introducing an inclined impinging flow, the mechanical scouring and stripping effects of the fluid on the gelled oil at the side wall are enhanced, breaking the thermal resistance barrier formed by the extremely low thermal conductivity of the crude oil gel layer. This mechanism is systematically demonstrated by establishing a broad-temperature-range mathematical model that characterizes the nonlinear thermophysical property variations during the gel–sol transition of waxy crude oil, combined with computational fluid dynamics simulations and laboratory-scale experiments. Field synergy analysis confirms that, under the 45° deflection condition, the proportion of regions with a synergy angle below 90° reaches 19.41%, significantly improving the coordination between the velocity field and the temperature gradient field. Benefiting from this synergistic effect and the intense wall scouring, the gelled crude oil achieves nearly complete sol-state conversion within 4 h, with the final conversion rate leaping to 99.75%, overcoming the disadvantages of standalone tubular heating, which yields a conversion rate of less than 35% and severe edge accumulation. Furthermore, compared to traditional global heating, the novel coupled process reduces the energy consumption per unit temperature rise by 12.78%. This study provides a new theoretical perspective and engineering guidance for energy conservation and solidification response strategies in high-pour-point crude oil storage and transportation systems.

## Graphical abstract

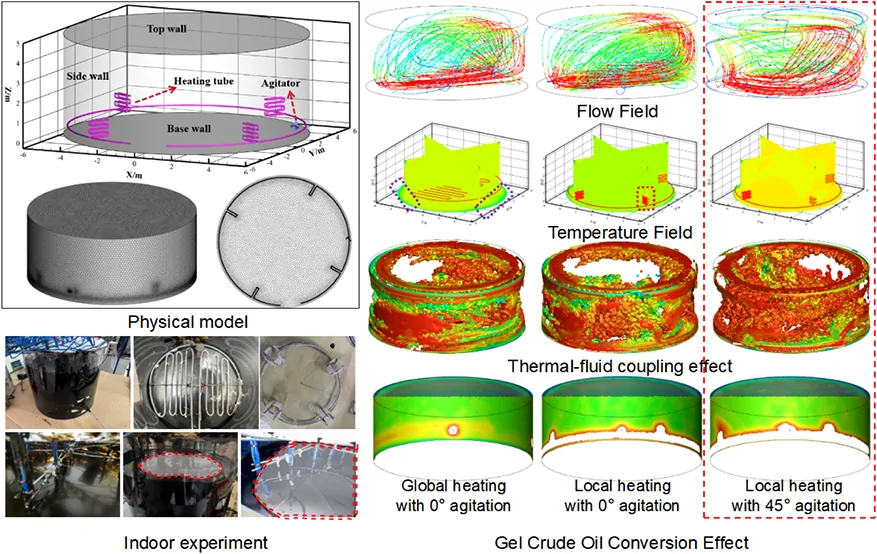

## 1 Introduction

Waxy crude oil is a key global resource, yet its physicochemical properties create persistent difficulties in extraction, transport, and storage.^1^ Below the wax appearance point, wax crystals precipitate and form a three-dimensional network, inducing a sol–gel transition and a drastic loss of fluidity.^2,3^ In storage tanks, continuous heat loss through the walls, floor, and roof lowers the oil temperature; when it falls below the pour point, the precipitated wax flocculates into a gel that entraps liquid oil, leading to solidification.^4–6^ This solidified oil reduces usable tank volume, clogs pipelines, hinders floating-roof movement, complicates restart operations, and may even cause safety accidents.^7^ Therefore, keeping the oil above the pour point or ensuring rapid and effective melting of gelled oil is essential for stable and safe storage-transport system operation.

In the management of solidified waxy crude oil, chemical methods (such as pour-point depressants, viscosity reduction via emulsification, and microbial treatment) and mechanical cleanup techniques^8–11^ (e.g., scraping mechanisms in floating-roof tanks) have been adopted. While effective to varying degrees, these approaches possess inherent limitations: chemical methods are constrained by temperature and reagent costs, whereas mechanical removal often necessitates tank shutdowns and suffers from efficiency constraints. By contrast, heating remains the most straightforward and widely utilized means for converting gelled oil. Conventional tank heating predominantly relies on globally arranged heating coils installed at the tank bottom, which transfer heat to various sections of the tank via natural convection.^12,13^ Nevertheless, this approach presents several drawbacks: first, the extremely low thermal conductivity of the gelled oil layer forms a substantial thermal barrier, impeding heat penetration to reach low-temperature dead zones at the tank bottom; second, in the gelled state, natural convection is suppressed, causing heat transfer to rely primarily on conduction, thereby reducing overall efficiency; third, the dispersed arrangement of heating coils results in insufficient local heat flux, failing to establish an effective temperature gradient within the gelled layer and consequently leading to substantial energy wastage.^14–16^ To mitigate these issues, mechanical agitation has been introduced as a heat transfer enhancement technique in storage tanks, aiming to disrupt thermal stratification through forced convection and promote uniform heat distribution,^17–21^ thereby eradicating solidified oil in low-temperature regions. Existing research has revealed that there exists an optimal range for impeller rotational speeds, beyond which the heating rate declines.^22^ Furthermore, different types of impellers generate distinct high-velocity core flows and recirculation patterns, leading to variations in heating performance.^23^ The impeller’s installation height and deflection angle also exert considerable influence on the elimination of solidified oil in critical low-temperature dead zones, such as those near the tank bottom and roof.^24^ In summary, current studies predominantly concentrate on the optimization of impeller geometry or operational speeds,^25–27^ whereas the critical aspect of “synergistic alignment” between the flow paths generated by agitation and the placement of heating sources remains insufficiently explored. If the impingement flow produced by stirring does not adequately cover the regions surrounding the heat sources or the areas of concentrated thermal energy, heating efficiency becomes sub optimal, consequently limiting the rate of solidified oil conversion. Additionally, waxy crude oil exhibits non-Newtonian behavior at low temperatures, with its viscosity being highly sensitive to variations in both temperature and shear rate. This characteristic significantly complicates the intertwined fluid flow and heat transfer processes inside storage tanks, imposing more stringent requirements on the design of stirring-heating integrated systems.

To address the aforementioned issues, this study, based on the traditional heating tube layout, proposed an improved configuration of local centralized heating tubes and, combined with a specific deflection angle of the agitator (in this study, 45° was selected as a representative angle for systematic research), aimed to establish a new heating process and achieve a reasonable matching between the flow path and the heat source arrangement. Using Daqing waxy crude oil as the working medium, a combined approach of numerical simulation and scaled laboratory experiments was employed to systematically investigate the flow and heat transfer characteristics within the storage tank under different heating-agitation configurations. Particular focus was placed on analyzing the velocity, temperature, and synergy angle distributions, as well as the effectiveness of gelled crude oil conversion, and the overall energy efficiency of the proposed system was evaluated. The findings presented herein are expected to provide valuable guidance for reducing energy consumption and ensuring the safe operation of heating systems in storage tanks containing high-pour-point crude oil.

## 2 Model Establishment

### 2.1 Construction of the Physical Model

#### 2.1.1 Construction of the Computational Domain

Taking a 1000 m^3^ floating-roof crude oil storage tank (Figure 1a) as the object, an improved local concentrated heating tube (Figure 1b) is proposed instead of the traditional overall layout (Figure 1c),^28^ and an agitator deflection angle of 45° (Figure 1d) is adopted. In this study, the 45° angle was initially determined based on engineering experience and the structure of the local heating tube. This 45° trajectory directs the agitator-induced flow directly into the core heat zone, enhancing convective heat transfer to remote dead areas and overcoming the high thermal resistance of the gelled layer, while also increasing the kinetic energy distribution and effectively scouring the wall gel, avoiding the common circumferential coverage gap at small angles (such as 30°) or the central area flow stagnation problem caused by large angles (such as 60°). It should be noted that in this study, continuous parameterization scanning was not conducted for the deflection angle, and 45° does not represent the globally optimal deflection angle. To balance accuracy and efficiency, a simplified model is used: the top, side, and bottom walls are treated as single-layer boundaries, with the total thermal resistance accounting for the underlying soil, side insulation, and top air layer, thus preserving the actual structural influence on internal temperature distribution (Figure 1).

**Figure 1:**
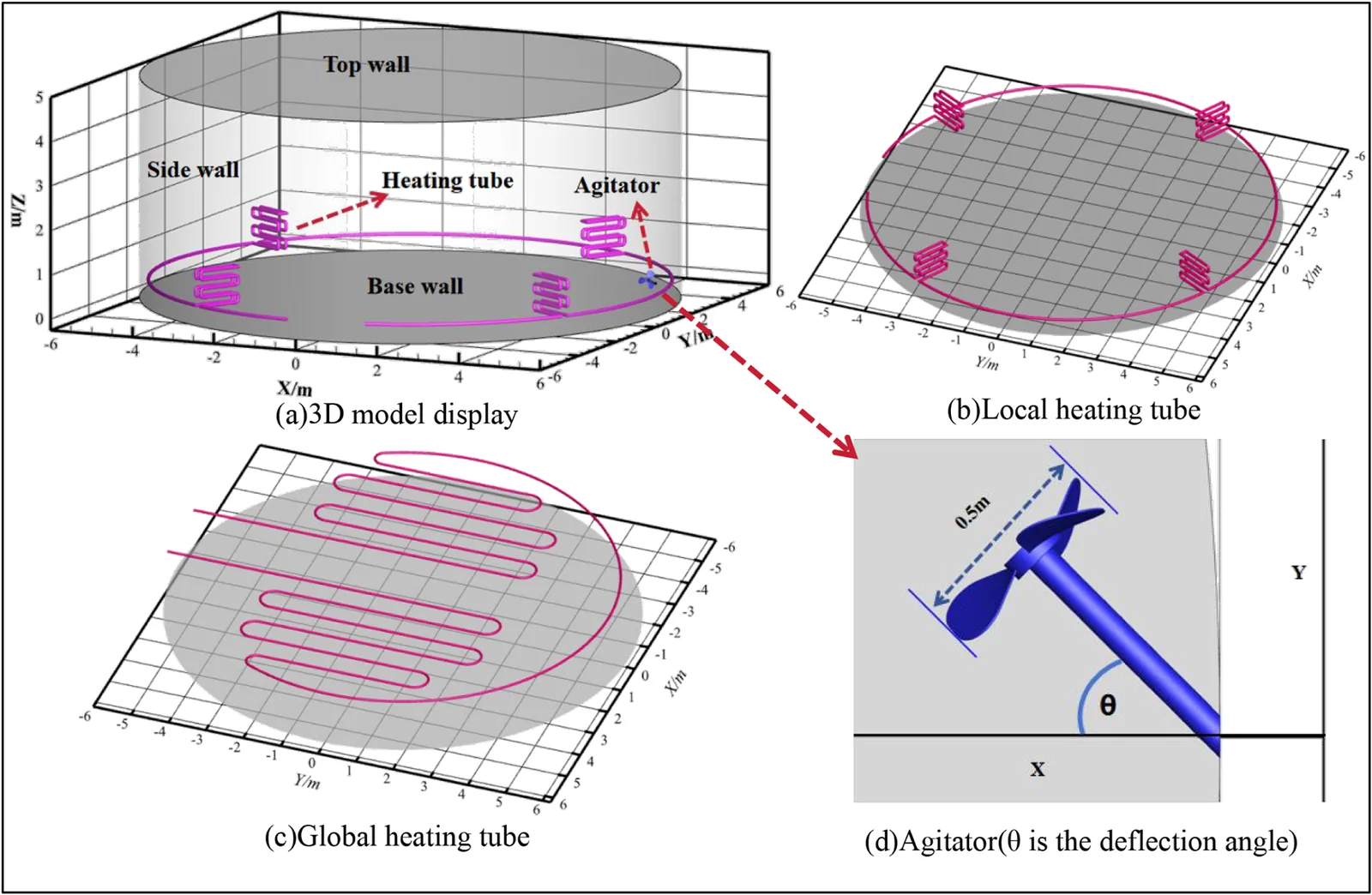
Physical model.

#### 2.1.2 Physical Properties of Crude Oil

Daqing waxy crude oil was selected as the simulation and experimental medium. The basic physical properties are shown in Table 1. Its thermophysical properties exhibit significant nonlinear characteristics with temperature variation.^29,30^ The viscosity-temperature relationship of the crude oil was measured using a QC rotational rheometer, as expressed by eq 1; the average error between the fitted values and the experimental values was 5.45%. Differential Scanning Calorimetry (DSC) experiments were conducted in accordance with the SY/T 0545–2012 standard, through which the wax precipitation temperature range was determined and the wax content was calculated. The temperature-dependent specific heat capacity was subsequently derived, with the results presented in Figure 2.

1 {μ=e3.82112−0.02165×Toil,50C°<Toil≤70C°μ=e6.96626−0.08153×Toil,40C°<Toil≤50C°μ=e(20.15303−0.3932)×Toil×γ−1.7988+0.04301×Toil,32C°<Toil≤40C°μ=e(13.5258−0.1694×Toil)γ+e10.1299817−0.10873×Toil×γ−0.52354+0.00902×Toil,10C°≤Toil≤32C°

**Table 1:** Properties of Daqing Wax Crude Oil

Density (20°C)	Heat conductivity coefficient (20°C)	Thermal diffusion coefficient (20°C)	Wax content	Viscosity (50°C)	Specific heat capacity (20°C)
870.1 kg/m^3^	0.175 W/m·°C	0.0753 mm^2^/s	25.3%	18.094 mPa·s	2918.1 J/kg·°C

**Figure 2:**
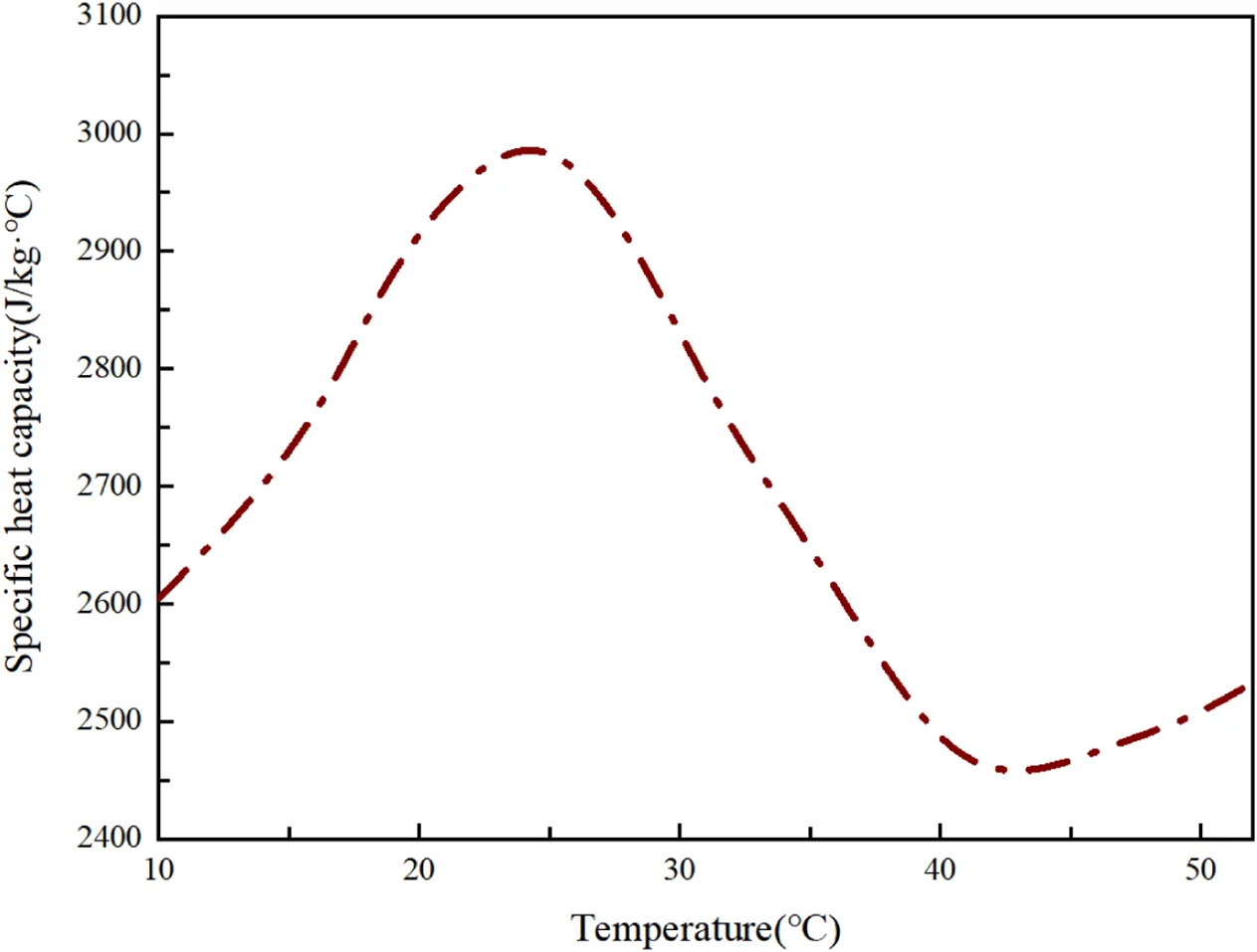
Variation curve of crude oil specific heat capacity with temperature.

## 3 Mathematical Models, Numerical Methods, and Grid Verification

### 3.1 Mathematical Models

#### 3.1.1 Basic Control Equations

The governing equations^31–34^ are presented in eqs 2–9.

2 $$\frac{\partial \rho }{\partial t}+\frac{\partial \left(\rho u\right)}{\partial x}+\frac{\partial \left(\rho v\right)}{\partial y}+\frac{\partial \left(\rho w\right)}{\partial z}=0$$

3 $$\begin{array}{ll} \frac{\partial \left(\rho u\right)}{\partial t}+\frac{\partial \left(\rho uu\right)}{\partial x}+\frac{\partial \left(\rho uv\right)}{\partial y}+\frac{\partial \left(\rho uw\right)}{\partial z} & =\frac{\partial }{\partial x}\left(\left(\mu +\mu_{t}\right)\frac{\partial u}{\partial x}\right)+\frac{\partial }{\partial y}\left(\left(\mu +\mu_{t}\right)\frac{\partial u}{\partial y}\right)+\frac{\partial }{\partial z}\left(\left(\mu +\mu_{t}\right)\frac{\partial u}{\partial z}\right)\\ & +\frac{\partial }{\partial x}\left(\left(\mu +\mu_{t}\right)\frac{\partial u}{\partial x}\right)+\frac{\partial }{\partial y}\left(\left(\mu +\mu_{t}\right)\frac{\partial v}{\partial x}\right)+\frac{\partial }{\partial z}\left(\left(\mu +\mu_{t}\right)\frac{\partial w}{\partial x}\right)-\frac{\partial P}{\partial x} \end{array}$$

4 $$\begin{array}{ll} \frac{\partial \left(\rho v\right)}{\partial t}+\frac{\partial \left(\rho vu\right)}{\partial x}+\frac{\partial \left(\rho vv\right)}{\partial y}+\frac{\partial \left(\rho vw\right)}{\partial z} & =\frac{\partial }{\partial x}\left(\left(\mu +\mu_{t}\right)\frac{\partial v}{\partial x}\right)+\frac{\partial }{\partial y}\left(\left(\mu +\mu_{t}\right)\frac{\partial v}{\partial y}\right)+\frac{\partial }{\partial z}\left(\left(\mu +\mu_{t}\right)\frac{\partial v}{\partial z}\right)\\ & +\frac{\partial }{\partial x}\left(\left(\mu +\mu_{t}\right)\frac{\partial u}{\partial y}\right)+\frac{\partial }{\partial y}\left(\left(\mu +\mu_{t}\right)\frac{\partial v}{\partial y}\right)+\frac{\partial }{\partial z}\left(\left(\mu +\mu_{t}\right)\frac{\partial w}{\partial y}\right)-\frac{\partial P}{\partial y} \end{array}$$

5 $$\begin{array}{ll} \frac{\partial \left(\rho w\right)}{\partial t}+\frac{\partial \left(\rho wu\right)}{\partial x}+\frac{\partial \left(\rho wv\right)}{\partial y}+\frac{\partial \left(\rho ww\right)}{\partial z} & =\frac{\partial }{\partial x}\left(\left(\mu +\mu_{t}\right)\frac{\partial w}{\partial x}\right)+\frac{\partial }{\partial y}\left(\left(\mu +\mu_{t}\right)\frac{\partial w}{\partial y}\right)+\frac{\partial }{\partial z}\left(\left(\mu +\mu_{t}\right)\frac{\partial w}{\partial z}\right)+\frac{\partial }{\partial x}\left(\left(\mu +\mu_{t}\right)\frac{\partial u}{\partial z}\right)\\ & +\frac{\partial }{\partial y}\left(\left(\mu +\mu_{t}\right)\frac{\partial v}{\partial z}\right)+\frac{\partial }{\partial z}\left(\left(\mu +\mu_{t}\right)\frac{\partial w}{\partial z}\right)-\rho_{20}\left(1‐\beta \left(T_{oil}-20\right)\right)g-\frac{\partial P}{\partial z} \end{array}$$

6 $$\frac{\partial \left(\rho T\right)}{\partial t}+\frac{\partial \left(\rho uT\right)}{\partial x}+\frac{\partial \left(\rho vT\right)}{\partial y}+\frac{\partial \left(\rho wT\right)}{\partial z}=\frac{\partial }{\partial x}\left(\frac{\mu }{{\mathrm{Pr}}_{t}}\frac{\partial T}{\partial x}\right)+\frac{\partial }{\partial y}\left(\frac{\mu }{{\mathrm{Pr}}_{t}}\frac{\partial T}{\partial y}\right)+\frac{\partial }{\partial z}\left(\frac{\mu }{{\mathrm{Pr}}_{t}}\frac{\partial T}{\partial z}\right)$$

7 $$\frac{\partial }{\partial t}\left(\rho k\right)+\frac{\partial }{\partial x}\left(\rho uk\right)+\frac{\partial }{\partial y}\left(\rho vk\right)+\frac{\partial }{\partial z}\left(\rho wk\right)=\frac{\partial }{\partial x}\left[\left(\mu +\frac{\mu_{t}}{\sigma_{k}}\right)\frac{\partial k}{\partial x}\right]+\frac{\partial }{\partial y}\left[\left(\mu +\frac{\mu_{t}}{\sigma_{k}}\right)\frac{\partial k}{\partial y}\right]+\frac{\partial }{\partial z}\left[\left(\mu +\frac{\mu_{t}}{\sigma_{k}}\right)\frac{\partial k}{\partial z}\right]+G_{k}-\rho \varepsilon$$

8 $$\frac{\partial }{\partial t}\left(\rho \varepsilon \right)+\frac{\partial }{\partial x}\left(\rho u\varepsilon \right)+\frac{\partial }{\partial y}\left(\rho v\varepsilon \right)+\frac{\partial }{\partial z}\left(\rho w\varepsilon \right)=\frac{\partial }{\partial x}\left[\left(\mu +\frac{\mu_{t}}{\sigma_{\varepsilon }}\right)\frac{\partial \varepsilon }{\partial x}\right]+\frac{\partial }{\partial y}\left[\left(\mu +\frac{\mu_{t}}{\sigma_{\varepsilon }}\right)\frac{\partial \varepsilon }{\partial y}\right]+\frac{\partial }{\partial z}\left[\left(\mu +\frac{\mu_{t}}{\sigma_{\varepsilon }}\right)\frac{\partial \varepsilon }{\partial z}\right]+C_{1\varepsilon }\frac{\varepsilon }{k}G_{k}-C_{2\varepsilon }\rho \frac{\varepsilon^{2}}{k}$$

The expression for *μ*_*t*_ is as follows:

9 $$\mu_{t}=\rho_{o}C_{\mu }\frac{k^{2}}{\varepsilon }$$

#### 3.1.2 Boundary Conditions

To simulate the boundary conditions of the actual storage tank, the following configurations were established based on its structural characteristics:^35,36^ The tank base wall is in contact with soil, beneath which lies a constant-temperature layer at a certain depth that remains unchanged throughout the year. Therefore, the first-type boundary condition is applied to the tank base wall (eq 10), with a constant temperature set at 5 °C, and the thermal resistance effect of the soil is incorporated into the thermophysical properties of the base wall material. The thickness of the soil layer below the tank base wall is set to 12.5 m. The side wall and top wall are directly exposed to the atmosphere, thus the third-type boundary condition is adopted (eqs 11–12), with an ambient temperature of −20 °C and a comprehensive heat transfer coefficient of 32.12 W/(m^2^·°C).^37,38^ For the roof boundary, the air layer and floating roof structure are considered, with an equivalent thickness of 0.7 m. For the wall boundary, the insulation layer is accounted for, with an equivalent thickness of 0.08 m. Specific boundary parameters^5,18,23,26,38^ are listed in Table 2. The heating tube is set as a first boundary condition (eq 13). The agitator only stirs, given a constant center of rotation and stirring (eq 14).

10 $$z=0,0\leq r\leq R_{\mathrm{tank}},T=T_{basewall},u_{i}=0$$

11 $$0\leq z\leq H_{\mathrm{tank}},r=R_{\mathrm{tank}},-\lambda_{sidewall}\left(\frac{\partial T_{sidewall}}{\partial x}+\frac{\partial T_{sidewall}}{\partial y}\right)=h_{sidewall}\left(T_{sidewall}-T_{air}\right),u_{i}=0$$

12 $$z=H_{\mathrm{tank}},0\leq r\leq R_{\mathrm{tank}},-\lambda_{topwall}\frac{\partial T_{topwall}}{\partial z}=h_{topwall}\left(T_{topwall}-T_{air}\right),u_{i}=0$$

13 $$T=T_{heat},u_{i}=0$$

14 $$-\lambda_{agitator}=\left(\frac{\partial T_{agitator}}{\partial n}\right)=0,x=x_{0},y=y_{0},z=z_{0},\tau =\tau_{0}$$

**Table 2:** Properties of Boundary Materials

Boundary	Density (kg/m^3^)	Thermal conductivity (W/m·°C)	(Specific heat capacity (J/kg·°C)
Top wall	1.225	1.05	1006
Side wall	60	0.055	800
Base wall	1600	1.74	1750

*x*_0_, *y*_0_, *z*_0_ are the rotation center coordinate of the agitator, *τ*_0_ is the mechanical stirring rate, rpm.

#### 3.1.3 Initial Conditions

15 $$T_{oil}\left(t=0,x,y,z\right)=T_{0},u\left(t=0,x,y,z\right)=v\left(t=0,x,y,z\right)=w\left(t=0,x,y,z\right)=0$$

### 3.2 Numerical Simulation Method

#### 3.2.1 Discretization of the Computational Domain

For an accurate characterization of the flow dynamics and heat transfer processes within the tank, the computational domain was partitioned into rotating and stationary regions using the sliding-grid methodology.^38^

Figure 3a and b, respectively, show the griding results of the overall structure and the base wall of the tank, a detailed grid configuration was implemented wherein local refinements of 6 mm and 10 mm were applied to the surfaces of the agitator (Figure 3c) and heating tubes (Figure 3d), respectively, while the tank top wall, side wall, and base wall were refined with a resolution of 180 mm. The maximum grid dimensions in the remaining regions were set to 200 mm. Data exchange between the rotating and stationary interfaces was facilitated through a conformal gridding approach, with the surface grid size uniformly maintained at 10 mm. Adopting this gridding strategy, the resultant grid system comprised approximately 2.86 million elements, with minimum and maximum cell sizes of 6 mm and 200 mm, respectively.

**Figure 3:**
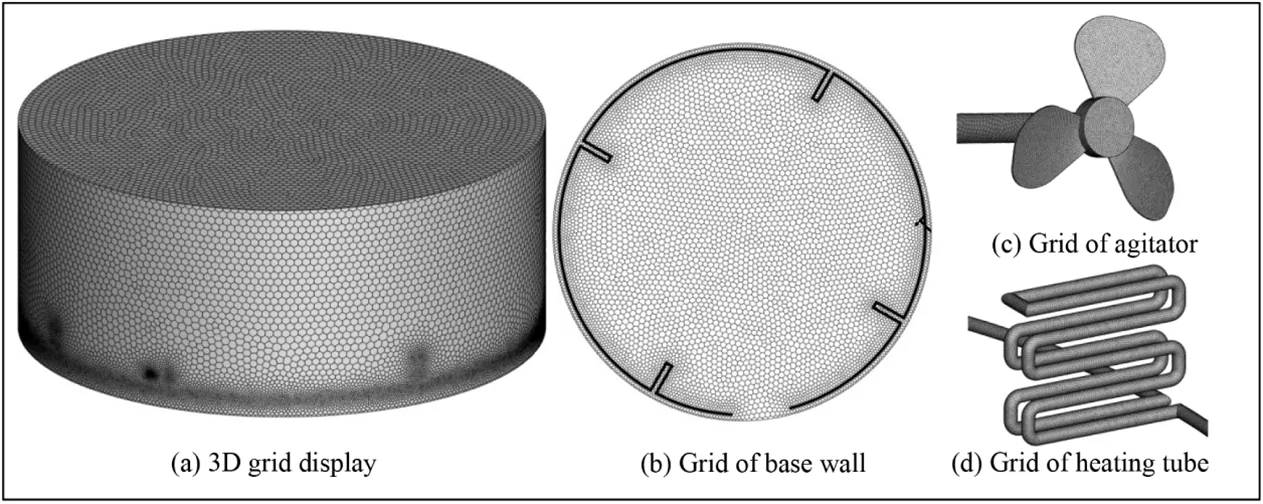
Details of grid division .

#### 3.2.2 Discretization of the Control Equations

The governing equations were discretized using the finite volume method. Specifically, the transient term was handled with a first-order fully implicit scheme, while the diffusion term was discretized by using a second-order central difference scheme. To improve computational accuracy, the convection term was treated by an improved QUICK scheme. For a more realistic representation of the pressure field distribution, a staggered grid strategy was adopted, and the pressure–velocity coupling was resolved via the SIMPLE algorithm. This algorithm operates on a staggered grid framework and iteratively solves the pressure field through a predictor-corrector approach, thereby significantly enhancing computational efficiency. Regarding the buoyancy effect induced by convection, the pressure term was discretized by using the volume-force weighting method to improve numerical stability. The turbulent kinetic energy and turbulent dissipation rate equations were discretized with a second-order upwind scheme. After constructing the algebraic equation system, the multigrid method was further employed to accelerate convergence and enhance solution efficiency.^39,40^

#### 3.2.3 Verification of Grid Independence

To mitigate the impact of grid discretization on the accuracy of numerical simulation results, a grid-independence verification was conducted. The specific content is shown in Table 3..

**Table 3:** Different Grid Division Schemes

Grid	Minimum grid size (mm)	Maximum grid size (mm)	Total count	Average temperature inside the tank at 900 s(°C)	*Nu* on the surface of heating tube after the calculation stabilizes
1	20	300	996090	32.0962	424.99
2	10	260	1924531	32.1040	548.16
3	6	200	2865012	32.1116	586.34
4	4	200	3223621	32.1134	599.56

As shown in Figure 4, as the grid is gradually refined, the average *Nu* on the heating pipe surface within the tank continuously increases. When the mesh density is increased from grid 1 to grid 2, the average temperature inside the tank rises by 0.24‰, the *Nu* increases by 28.98%, the number of meshes increases by 93.21%, and the calculation time increases by 41.38%. Further refining to grid 3, the average temperature inside the tank rises by 0.22‰, the *Nu* increases by 6.97%, the number of meshes increases by 48.87%, and the calculation time increases by 26.83%. After refining to grid 4, the average temperature inside the tank only rises by 0.06‰, the *Nu* only increases by 2.25%, the number of meshes increases by 12.52%, and the calculation time increases by 13.46%. From the above results, Grid 3 was selected as the optimal solution.

**Figure 4:**
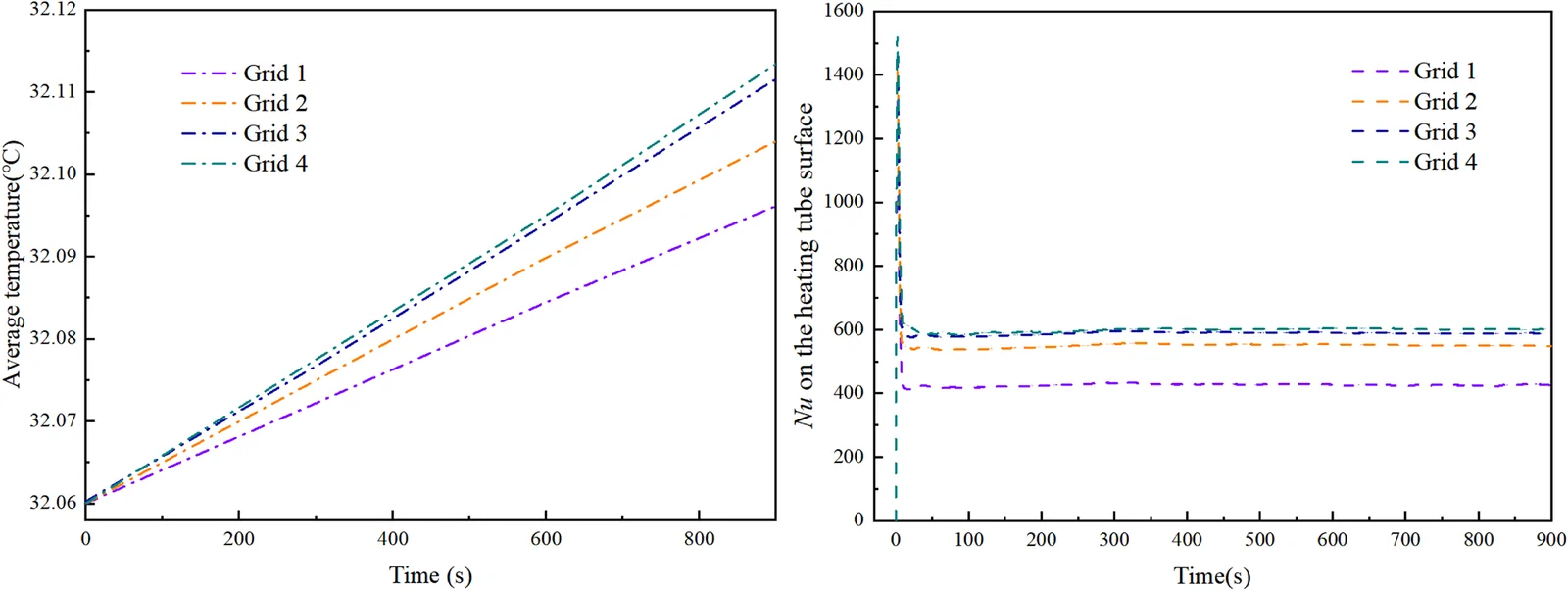
Variation of average temperature in the tank and *Nu* on the surface of the heating tube under different grid conditions.

## 4 Experimental Verification

### 4.1 Experimental Equipment

Scaled laboratory tests were performed to validate the numerical model. The setup comprised a small tank (outer diameter of 36.0 cm, wall thickness of 0.5 cm, height of 30.0 cm) filled with crude oil to 24.0 cm (Figure 5a). Both global and local heating tubes (Figure 5b–c) were made of rubber hoses with equal total length. The agitator was scaled to the simulation dimensions and installed at 5.0 cm height, while the heating tubes were at 1.0 cm. Daqing crude oil was used. Temperature sensors (Figure 5d) recorded data at 10-s intervals, with placements shown in Figure 5e–f.

**Figure 5:**
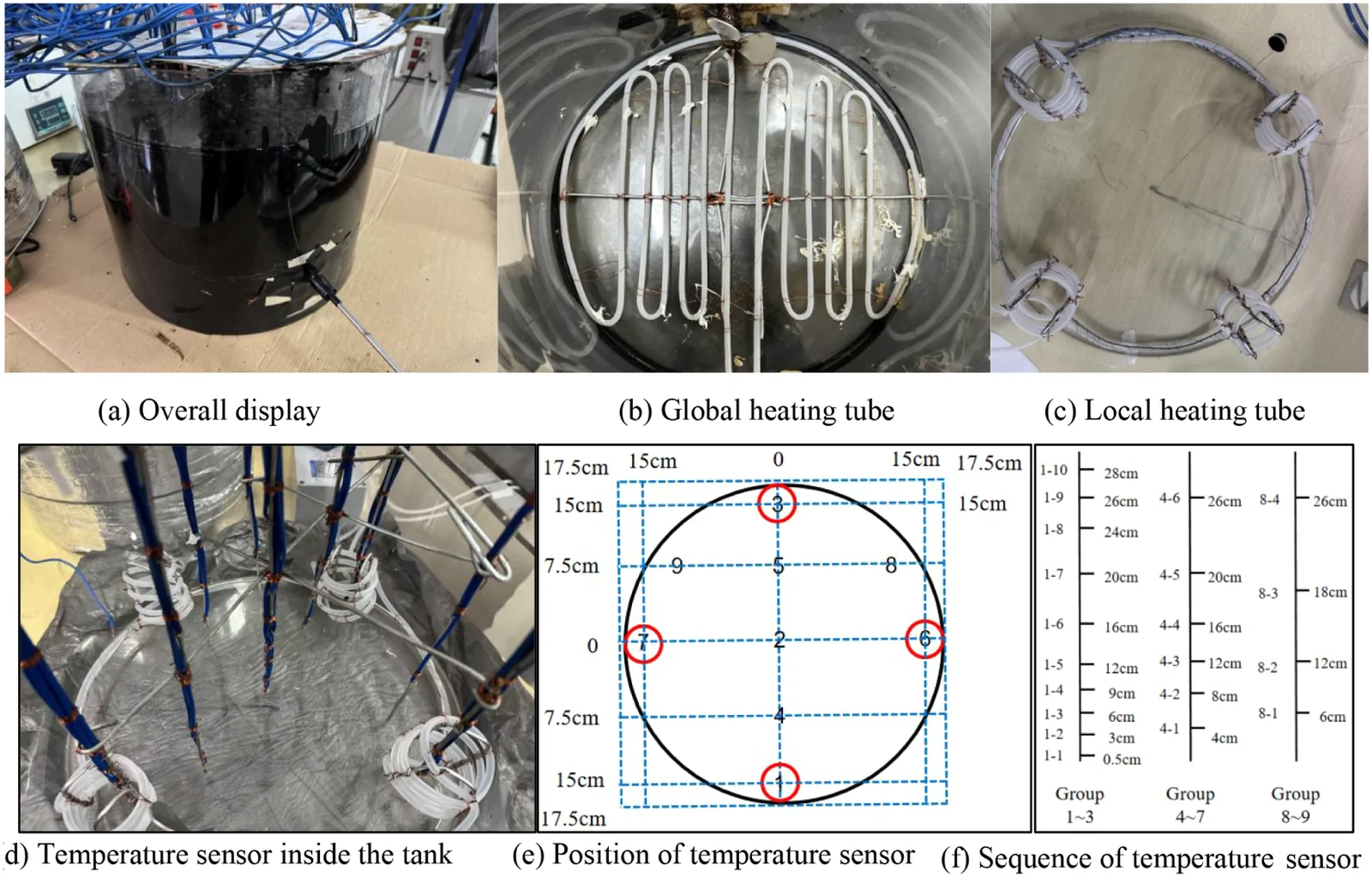
Experimental equipment.

### 4.2 Validation Experiments

Utilizing the aforementioned experimental equipment, validation was conducted through local heating tube combined with a 45°-deflection agitation-heating experiment. Simultaneously, a numerical simulation model was constructed at a 1:1 scale based on the mathematical model (Figure 6), gridding strategy, and numerical algorithms employed in this study, Figure 6a shows the overall view of the tank and Figure 6b and c, respectively, show the details of the agitator and the heating tube. Corresponding numerical simulations were performed under the same operating conditions. By comparing critical physical parameters obtained from both experiments and simulations, the accuracy of the numerical simulation methodology adopted in this work was effectively verified.

**Figure 6:**
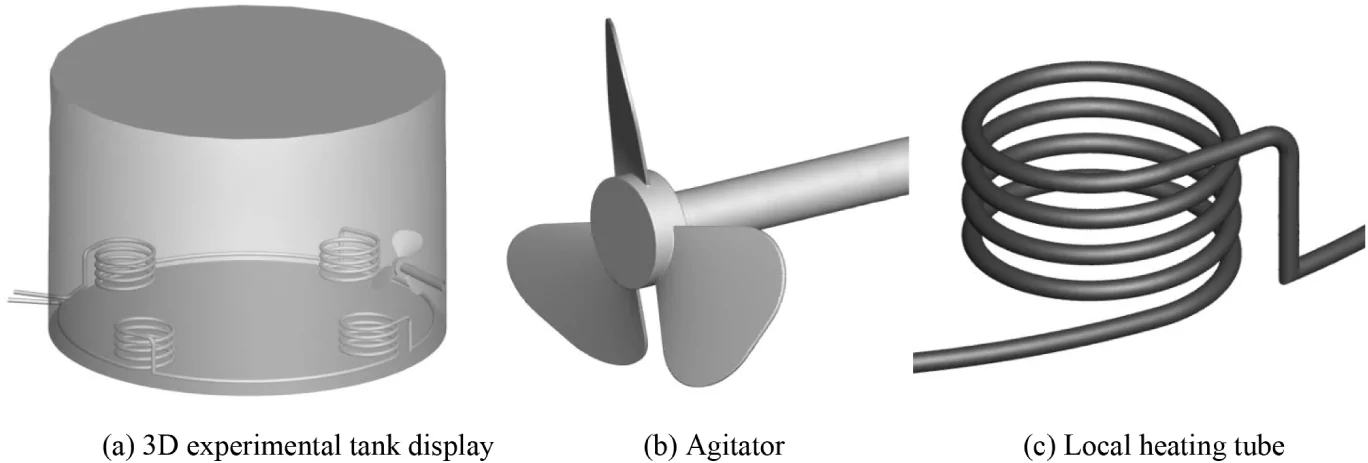
Simulation model display.

The temperature sensors were calibrated prior to the experiment. The tank was placed in a 50 °C constant-temperature chamber for 2 h, after which 50 °C hot water was injected to verify all sensor readings; all errors were within 0.2 °C. During the experiment, the crude oil was cooled from 40 °C to an average of 32 °C, then heating, combined with 45° deflection agitation, was initiated, with the heating tube inlet maintained at 75 °C. Figure 7 compares the measured and simulated temperature variations at characteristic points after 1 h of heating.

**Figure 7:**
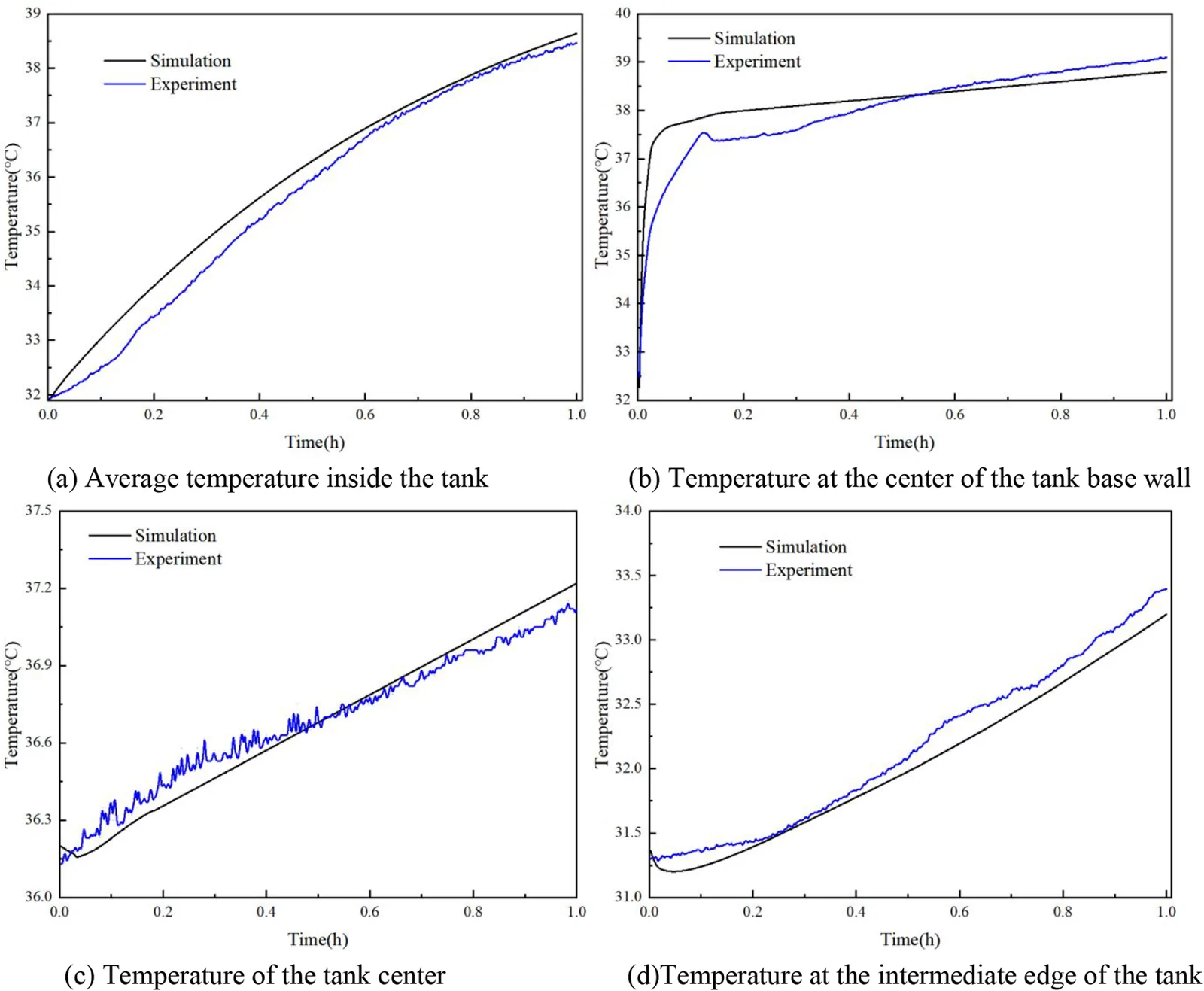
Comparison of experimental and numerical simulation results.

Given the unavoidable discrepancies between experimental and simulation conditions, along with uncontrollable factors, some deviations are expected. The main causes include limited instrument accuracy (mitigated by increased sensor density), small fluctuations in the actual ambient temperature (modeled as constant and addressed by selecting stable periods and using average temperatures), and minor disturbances from personnel movement and lighting variations. These factors are estimated to affect the overall results by less than 1%.

Figure 7 presents a comparative analysis of temperature variations between numerical simulations and experiments over the course of one hour of heating. The comparisons encompass the average internal tank temperature (Figure 7a), as well as temperature trends at the center of the tank base wall (Figure 7b), the center of the tank (Figure 7c), and the intermediate edge of the tank (Figure 7d). In terms of the average internal temperature after one hour of heating, the overall trends observed in numerical simulations and laboratory experiments for the small-scale storage tank show substantial agreement. The average heating rates recorded in the numerical simulation and the experiments are 6.71 °C/h and 6.52 °C/h, respectively, corresponding to an error of 2.88%. The trends for temperature variations at the base wall center, tank center, and intermediate edge of the tank are also consistent between numerical simulation and experimental data. Statistical calculations indicate that the maximum error does not exceed 4.51%, while the overall average relative error stands at 1.58%, which satisfies the required precision thresholds. The above results demonstrate that the numerical simulation method adopted in this study is reliable and has good computational accuracy. These results lay a theoretical and experimental foundation for subsequent parametric numerical simulations and ensure the accuracy of related research. Furthermore, the numerical simulation method we have adopted is also applicable to larger storage tanks.^18^

In order to further verify the essential similarity between the small-scale experiments and the large-scale numerical simulations in terms of fluid mechanics and heat transfer, this study introduces a dimensionless comparison analysis of the Prandtl number (*Pr*) and the Richardson number (*Ri*; usually, when *Ri* < 0.1 natural convection can be ignored, when *Ri* > 10 forced convection can be ignored, and when 0.1 < *Ri* < 10 it belongs to mixed convection, that is, both natural convection and forced convection cannot be ignored).

16 $$\mathrm{Pr}=\frac{\mu c_{p}}{k}$$

17 $$Ri=\frac{g\beta \Delta TL}{v^{2}}$$

where *μ* represents the dynamic viscosity of the fluid inside the tank, measured in Pa·s; *c*_*p*_ is the specific heat capacity of the fluid, measured in J/(kg·°C); *k* is the thermal conductivity, measured in W/(m·°C); *g* is the gravitational acceleration, 9.8 m/s^2^; *β* is the volumetric thermal expansion coefficient of the fluid, measured in °C^–1^; *ΔT* is the characteristic temperature difference, measured in °C; *L* is the characteristic length, measured in m; *v* is the characteristic flow velocity, measured in m/s.

According to the eq 16, *Pr* is only related to the thermophysical properties of the fluid itself. For the indoor experiment and the 1000 m^3^ industrial tank, they use the same Daqing waxy crude oil and their physical properties are the same at the same temperature. Therefore, *Pr* is also equal in the two scales. At the same time, we calculated the Richardson number (*Ri*) inside the tank (eq 17). The calculated *Ri* value inside the 1000 m^3^ tank is 3.21×10^–3^ ≪ 0.1, and the *Ri* value inside the indoor experiment tank is 1.95×10^–3^ ≪ 0.1. Both are far less than 0.1 (when *Ri* < 0.1, natural convection can be ignored), indicating that the state inside the tank at this time is in forced convection. The above results comprehensively indicate that the laboratory test tank and the simulated 1000 m^3^ tank satisfy the key similarity criteria.

## 5 Results and Discussion

### 5.1 Numerical Simulation Results

#### 5.1.1 Summary of Simulation Cases

The specific simulation cases are detailed in Table 4 below.

**Table 4:** Simulation Cases

case	Heating method	Deflection agitation of agitator	Velocity of agitation (RPM)	Temperature of heating tube (°C)
1	Only global heating	/	/	70
2	Only local heating	/	/	70
3	Global heating with agitation	0	350	70
4	Local heating with agitation	0	350	70
5	Local heating with agitation	45	350	70

#### 5.1.2 Temperature Field Analysis

The critical physical parameters within the storage tank during the heating process (average temperature and average temperature variance) were initially extracted, as presented specifically in Figure 8 below.

**Figure 8:**
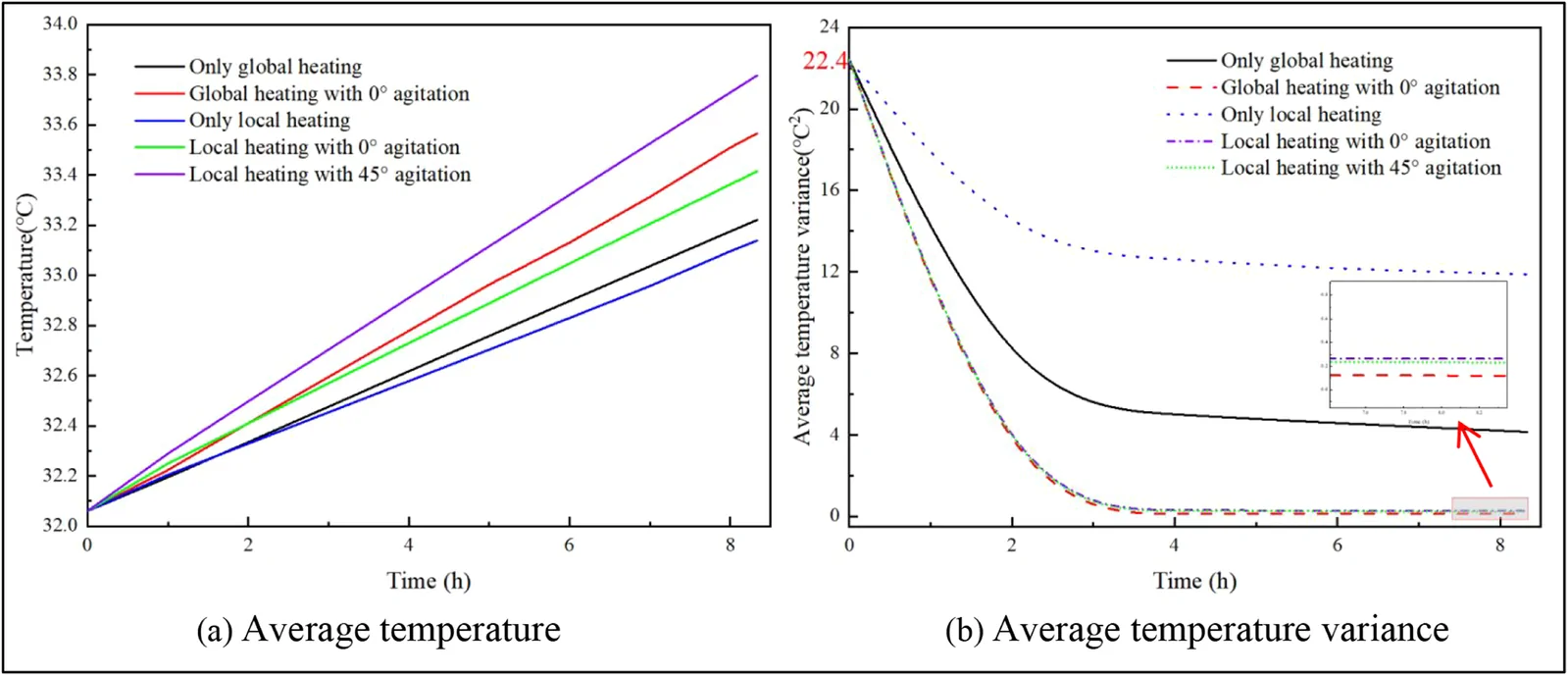
Changes in physical indicators inside the tank during the heating process.

Under conditions of only global heating and only local heating, respectively, the average temperature rises slowly, with tube-dependent effects. The initial temperature field had a variance of 22.4 °C^2^, indicating extremely poor uniformity. After 8.3 h of heating, the averages are 33.22 °C and 33.14 °C, with variances reduced to 4.13°C^2^ and 11.87 °C^2^, respectively, still showing poor uniformity. Variance drops sharply in the first 2 h (by 75.22% and 41.57%) and then stabilizes, indicating that tube-only heating insufficiently melts gel oil in dead zones; prolonged operation worsens adverse impacts.

After the agitation effect is introduced, the strong impinging flow generated by the agitator influences the entire computational domain. The temperature variation induced by the heating tubes begins to intensify with the impact of the agitator, and the high-temperature zones formed around the heating tubes are gradually disrupted by the agitation action. These effects significantly suppress the local characteristics of the heating tubes and directly influence the temperature indicators within the tanks. Moreover, distinct differences are observed in the heating efficiency under varying synergistic angles between the different heating tubes and the deflection angles. In terms of the average temperature (Figure 8a), the heating performance with synergistic agitation is notably superior to that of only tubular heating.

Overall, among the five different heating methods studied, local heating with 45° deflection agitation exhibited higher heating efficiency. After 8.3 h, the average tank temperature rises to 33.80 °C, exceeding those of only global heating, only local heating, global heating with 0° agitation, and local heating with 0° agitation by 0.58 °C, 0.66 °C, 0.23 °C, and 0.38 °C, respectively. The average heating rate is approximately 0.21 °C/h, representing a 49.64% and 61.04% increase compared to only global and local heating, respectively, and a 15.33% and 28.17% increase relative to global heating with 0° agitation and local heating with 0° agitation, respectively. This enhancement is attributed to the intense impinging flow generated by the 45° deflected agitator, which directly impacts the high-temperature crude oil near the localized heating tubes; the oil is then dispersed circumferentially and along the wall surfaces, promoting rapid melting of the gelled crude oil at the base, side, and top walls. Additionally, after incorporating mechanical agitation, the temperature variance reduction follows a “fast initial, gradual later” trend. After 2 h of heating, the temperature variances under different heating methods decrease to approximately 0.3 °C^2^—a reduction of over 98% compared to the initial state—indicating high temperature field uniformity by that point.

The initial temperature field (Figure 9a) developed after cooling from a uniform 40 °C to an average of 32.06 °C (near the gel point of 32 °C), resulting in a non-uniform distribution. Low-temperature zones (blue) appear near the base, side, and top walls, where the temperature falls below the gel point, causing gelled crude with poor fluidity and high thermal resistance. A vertical temperature gradient exists from the base to the top in the central region, and the overall uniformity is very poor, with a variance of 22.4 °C^2^. This condition resembles actual tank solidification due to prolonged shutdowns or heat loss, highlighting the need for an efficient heating method.

**Figure 9:**
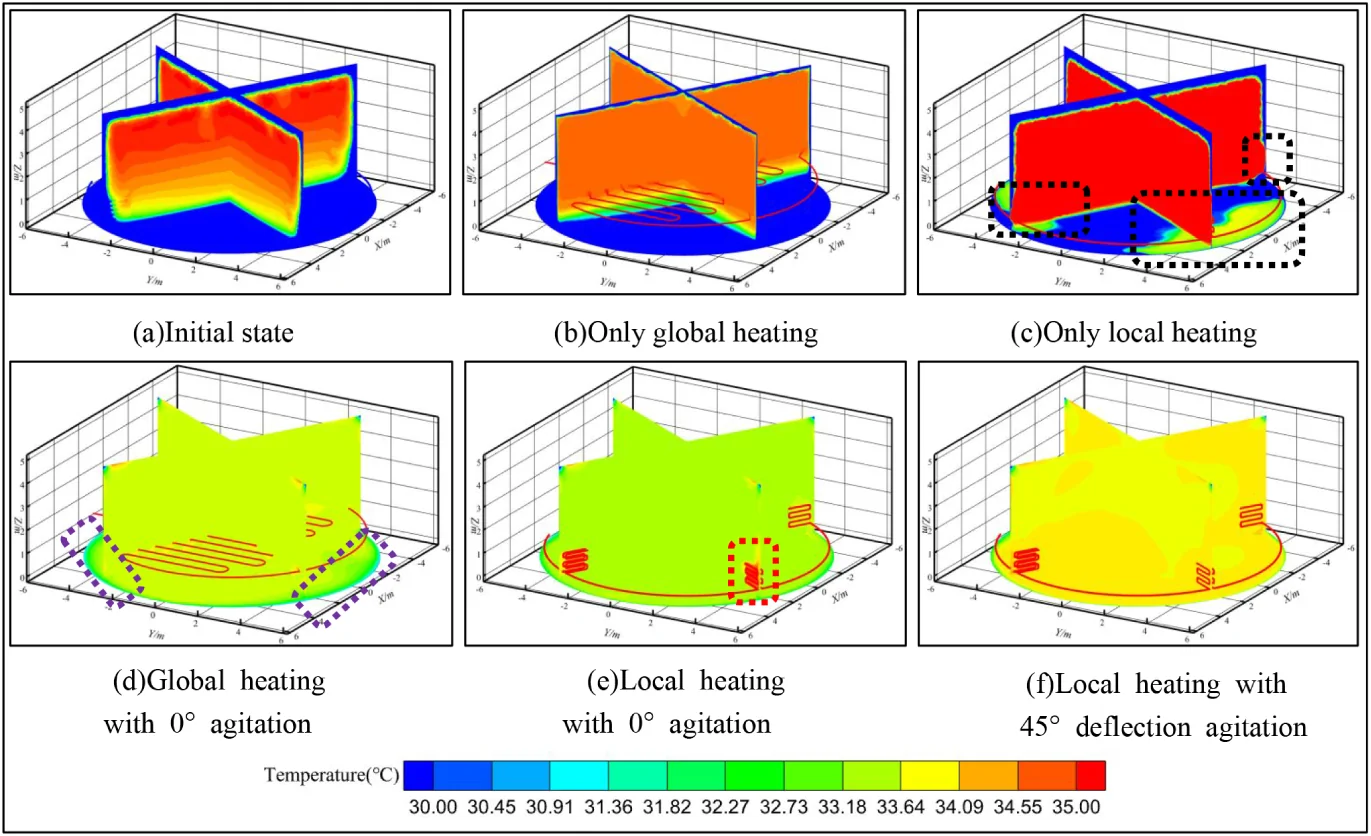
3D temperature field cloud maps after 8.3 h of heating.

Figure 9 shows that under tube-only heating, global heating (Figure 9b) outperforms local heating (Figure 9c). Uniform global tubes raise temperatures across most areas, reducing initial low-T zones near the side/top walls, though most remain below the gel point. At the base wall, weak convection and dispersed tubes limit heating, so the dead zone warms little. Uniformity improves in non-boundary regions (orange areas) vs. initial, with a final variance of 4.14 °C^2^. In contrast, local heating (Figure 9c) causes intense warming near the concentrated zone (black-bordered area), reducing gelled oil, but remote regions (upper side wall, top wall, central base) warm minimally; the top-wall low-T zone expands. The final variance 11.874 °C^2^, much higher, indicating lower efficiency alone. Overall, neither alone suffices to eliminate low-T areas or melt most gelled crude.

The introduction of mechanical agitation significantly improves the overall condition, yet the rational matching between the flow path and the heating source is equally crucial. By comparing the overall temperature field cloud maps of the three heating methods combined with mechanical agitation, it is evident that the configuration with localized heating with 45° deflected agitation (Figure 9f) yields the most effective heating outcome. Under this setup, the overall temperature within the tank is higher, and the initial low-temperature zones (below 32°C) are almost entirely eliminated, resulting in the near-complete transformation of gelled crude oil in these areas into a sol state. In contrast, when global heating is employed with 0° agitation (Figure 9d), the impinging flow propagates axially in a straight path and makes segmented contact with the high-temperature crude oil near the heating tubes. This leads to two issues: First, due to the segmented contact with the heating tubes, the overall temperature of the high-temperature crude oil carried by the impinging flow remains relatively low. As the flow advances, heat dissipates gradually, resulting in insufficient thermal energy upon reaching low-temperature dead zones such as the tank base wall, side wall, and top wall. Consequently, the crude oil in these regions is not fully melted, leading to sub optimal overall heating and gel-melting efficiency. Second, the straight and forward advance of the impinging flow will lead to local low temperatures in the area far from the impinging flow path in the tank, resulting in an obvious temperature gradient (within the purple frame line in the cloud map). The persistence of these low-temperature dead zones further limits the efficiency of gel conversion. Similarly, when localized heating is combined with 0° agitation (Figure 9e), although the crude oil near the concentrated heating zone reaches a higher temperature, the impinging flow generated by the agitator fails to effectively interact with the high-temperature region. This mismatch leads to inadequate overall heating performance, as clearly indicated by the distinct local heating tube signatures visible in the temperature contours (marked by red dashed boxes). In contrast, localized heating with 45° agitation reasonably aligns the flow path with the heat source. The 45° deflected flow transports hot oil from the heating tubes to low-temperature zones (e.g., base and side walls), enhancing temperature rise and accelerating gelled oil conversion. Further analysis of the base-wall and top-wall temperature fields is presented below.

At the initial moment (Figure 10a), the average temperature at the tank base wall was only 27.07 °C, with the maximum also below 32.0 °C, indicating that the crude oil had completely gelled. Under global heating alone (Figure 10b) and local heating alone (Figure 10c), the average base wall temperatures were 24.52 °C and 30.13 °C, respectively, with heat dissipation exceeding heat input in the global case, causing further temperature decline. Although local heating induced some temperature rise, the localized characteristics of the heating tubes were overly pronounced (Figure 10), resulting in highly uneven temperature distribution and a low average heating rate of only 0.36 °C/h. With mechanical agitation, the base wall temperature field improved significantly, especially for local heating with 45° deflection agitation (Figure 10f), where only the edges showed slightly lower temperatures. By the final moment, the average base wall temperature reached 33.79 °C, compared to 33.39 °C for global heating with 0° agitation (Figure 10d) and 33.36 °C for local heating with 0° agitation (Figure 10e), aligning closely with the overall tank average, and the average heating rate was 0.81 °C/h. More importantly, the temperature difference between the maximum (34.0 °C) and minimum (31.52 °C) was 2.48 °C, lower than that for global heating with 0° agitation (2.81 °C, max 33.60 °C, min 30.79 °C) and local heating with 0° agitation (3.48 °C, max 33.50 °C, min 30.02 °C), demonstrating superior temperature uniformity at the base wall and more rapid transformation of gelled crude oil in low-temperature zones.

**Figure 10:**
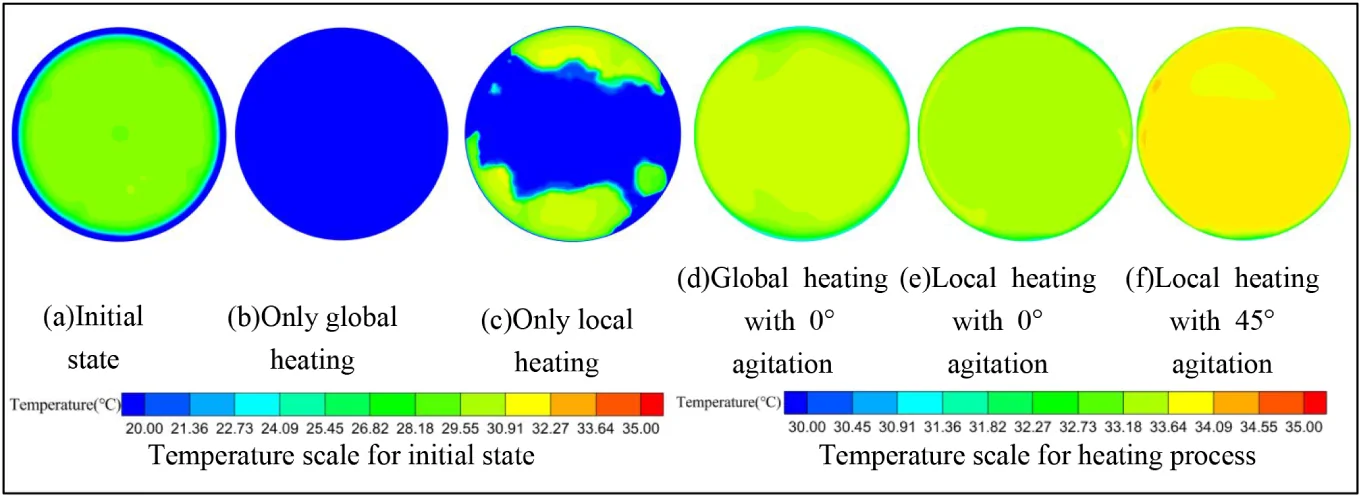
Cloud map of the temperature field at the base wall after 8.3 h of heating.

The initial average temperature (Figure 11a) at the tank top wall is 5.1 °C, with the crude oil therein in a gelled state. Under both independent global heating (Figure 11b) and local heating (Figure 11c) conditions, the heat dissipation of the crude oil in this area exceeds the thermal energy acquired, resulting in a final temperature remaining below 10.0 °C. With the application of mechanical agitation combined with heating, the temperature distribution cloud map of the tank top wall distinctly reveals a significant temperature elevation. In this scenario, the thermal characteristics exhibit a low-temperature zone at the outermost periphery (represented by the blue area in Figure 11, with temperatures approximating 30.0°C), followed immediately adjacent to the inner tank edge by a high-temperature band (exceeding 34.0°C), and subsequently, other regions within the medium-high temperature range. Comparative analysis indicates that the three-part differential under global heating with 0° agitation (Figure 11d) is more pronounced (with a temperature spread of 9.42 °C between the highest and lowest), followed by local heating with 0° agitation (Figure 11e,differential of 8.14°C). In contrast, the most uniform profile is achieved through local heating with 45° deflected agitation (Figure 11f,differential of 7.62°C). Moreover, the low-temperature peripheral zone under this latter regimen is observed to be reduced in extent relative to the other two heating configurations, thereby enhancing the melt efficiency of gelled crude oil at the tank top within an equivalent time frame.

**Figure 11:**
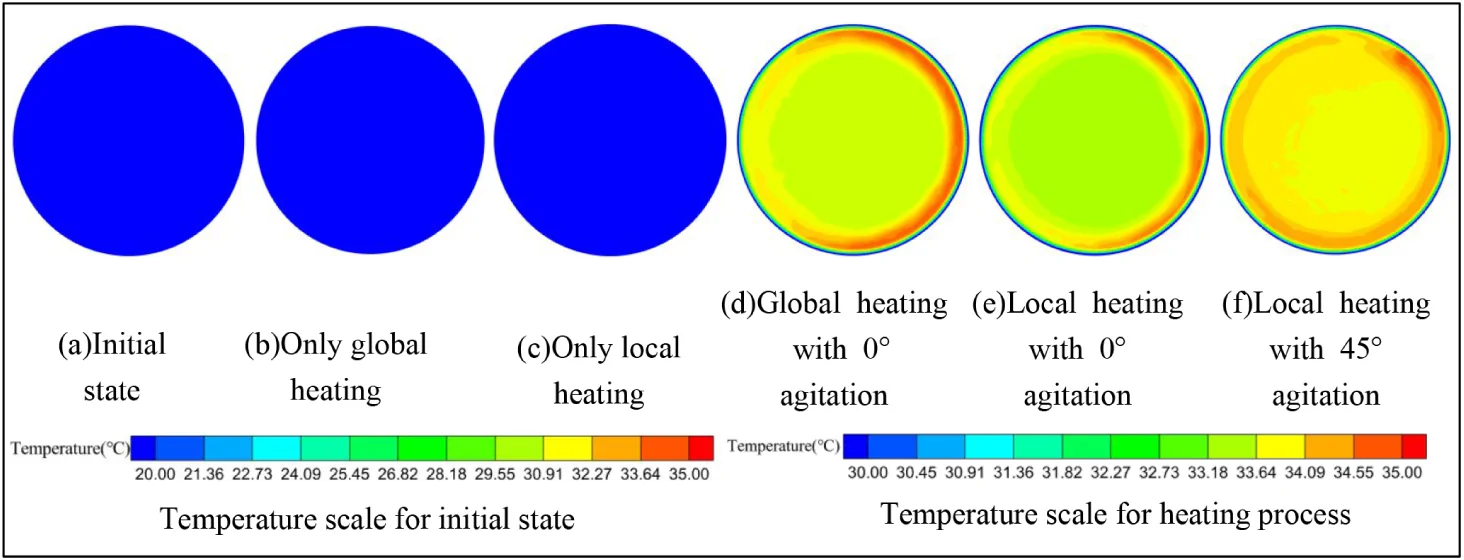
Cloud map of the temperature field at the top wall after 8.3 h of heating.

Based on comprehensive temperature data and cloud maps, without agitation, both global and local heating suffer from slow rates, poor uniformity, pronounced top–bottom stratification, and persistent dead zones—deficiencies more severe for local heating. With agitation, overall patterns remain similar across heating types and deflection angles, but local heating with 45° deflection consistently yields better average temperature rise and field homogeneity. The 45° inclined jet continuously transports heat from the tubes to other regions, enhances convection near heated surfaces, and, together with the annular local heater, effectively mitigates base wall dead zones common in traditional methods. In practice, conventional global heating cannot rapidly melt gelled crude during tank solidification or extended shutdowns, and efficiency drops as the gel layer thickens. In contrast, the proposed local heating with a deflected agitator (exemplified by the 45° case in this study) can quickly establish forced circulation: the inclined impinging jet mechanically disrupts the gel structure while carrying concentrated heat throughout the tank, forming more effective convective channels compared with the 0° axial-agitation case. As the gel melts and fluidity recovers, the flow field expands toward the side and top walls, notably improving the melting efficiency under this configuration.

#### 5.1.3 Flow Field Analysis

The temperature field analysis indicates that among the five different heating methods, the overall heating efficiency achieved by combining local heating with 45° deflection agitation is higher than that of the traditional heating method. The evolution of the temperature field is fundamentally determined by variations in the flow field. The following section will analyze the flow field distribution characteristics of each heating method. Figure 12 illustrates the streamline cloud maps depicting the motion of crude oil within the tank under different heating methods.

**Figure 12:**
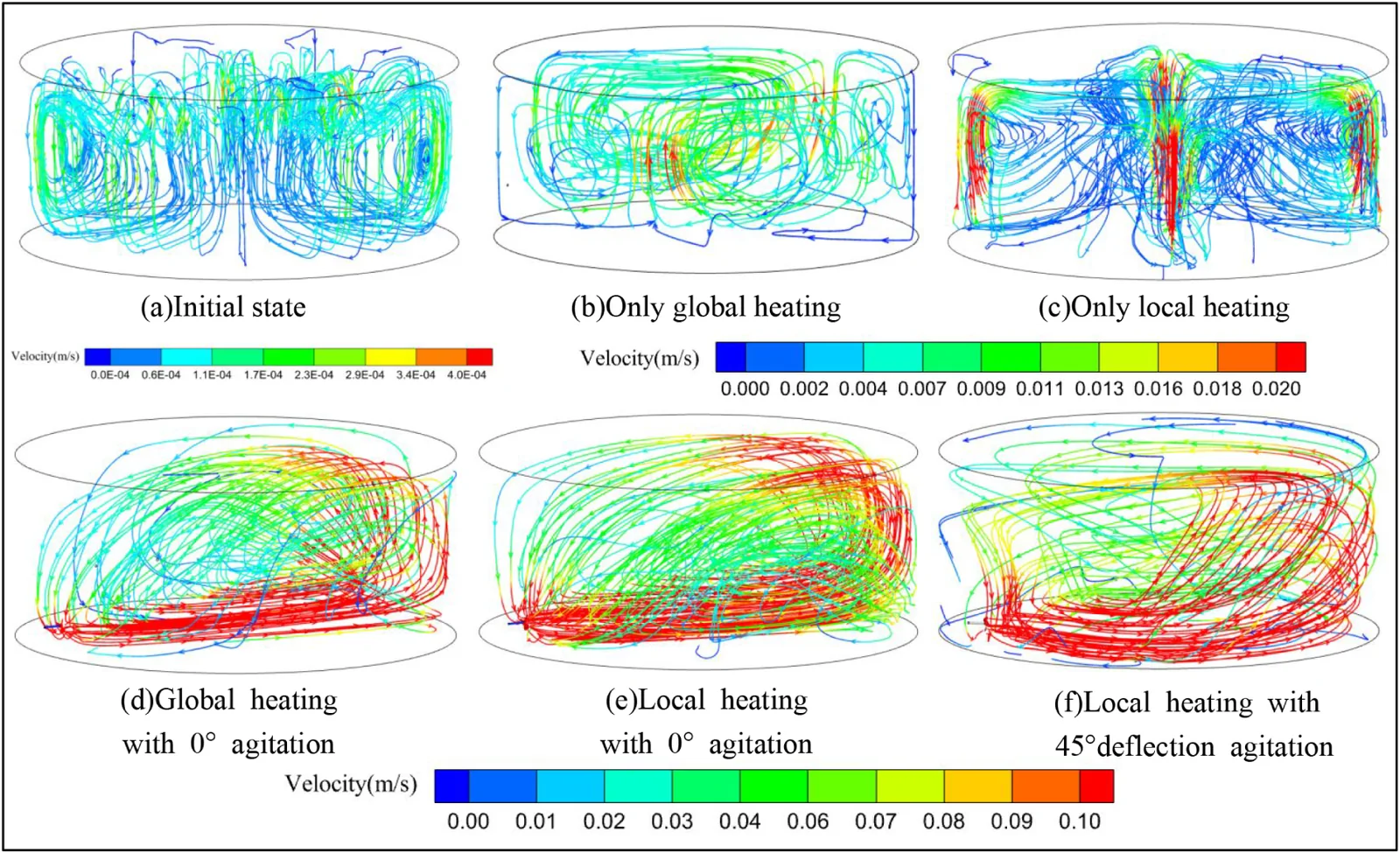
Streamline cloud maps depicting the motion of crude oil within the tank.

During the cooling process (Figure 12a), the flow is extremely weak, with minimal natural convection. Under global heating alone (Figure 12b), the flow intensifies in the central region due to a higher initial oil temperature and enhanced temperature differences, strengthening natural convection. With local heating (Figure 12c), the flow concentrates directly above the heating zone. Overall, the flow remains weak in both tube-heating cases, with large stagnant areas near the base and top walls (consistent with the temperature fields), leading to gelation in those regions.

When mechanical agitation is combined with heating, the flow within the tank becomes notably intensified, leading to a corresponding enhancement of heat transfer. For both global heating with 0° agitation (Figure 12d) and local heating with 0° agitation (Figure 12e), the primary flow trajectories are essentially similar, manifesting as unidirectional impinging flows along the axial direction: the flow propagates linearly along the agitator axis, impacts the opposite side wall, ascends vertically along the wall surface, and returns along the top wall to the agitator, forming a unidirectional circulation. Areas far from the agitator axis, including portions of the base wall, side walls, and top wall, remain largely unaffected with relatively weak flow. In terms of flow velocity, global heating with 0° agitation exhibits a weaker flow (average velocity of 0.047 m/s) compared to local heating with 0° agitation (0.059 m/s). This attenuation is attributed to the uniformly distributed heating tubes at the tank base, which impose resistance on the impinging flow, causing kinetic energy loss before it reaches the opposite wall and shortening the upward development and the reflux interval along the wall. In contrast, local heating with 0° agitation experiences negligible kinetic energy loss in this regard, resulting in more intense flow reaching the top wall with broader reflux coverage, consistent with the top-wall temperature distribution in Figure 11. However, 0° agitation presents two primary limitations: the flow trajectory is relatively simple, and the horizontal impingement against the tank wall causes kinetic energy loss while imposing considerable load on the tank structure. Employing local heating with 45° deflection agitation (Figure 12f) effectively addresses these issues. The flow intensity (average velocity of 0.068 m/s) surpasses that of the two 0° cases, and the flow trajectories divide into two distinct directions: one involves impact against the opposite wall followed by spiral ascent to the top wall, with notably stronger and more extensive flow activity covering a larger area of the top wall; the other follows the peripheral circumference along the wall post-impact, extending to regions inaccessible under 0° agitation and alleviating mechanical load on the side wall. Consequently, thermal energy from the heating tubes can be effectively conveyed to low-temperature dead zones such as the top and base walls, enabling adequate phase transition of gelled crude oil in these areas.

Another key advantage of localized heating with 45° agitation is the rational alignment (Figure 13) between flow trajectories and heat sources (This does not mean that 45° is the optimal deflection angle). Traditional global heating with 0° agitation (Figure 13a) has dispersed heat sources and insufficient concentration. Local heating with 0° agitation (Figure 13b) provides concentrated heat but suffers from flow-path mismatch. Under 45° agitation (Figure 13c), the high-velocity core flow directly impinges on the dense heating-tube zone, transporting hot oil throughout the tank. The streamline distribution confirms that agitation covers most areas, effectively scouring and reducing gelled oil and sediment at low-velocity zones (e.g., base wall), while the carried heat helps melt accumulated oil at both the base and the top, eliminating low-temperature dead zones.

**Figure 13:**
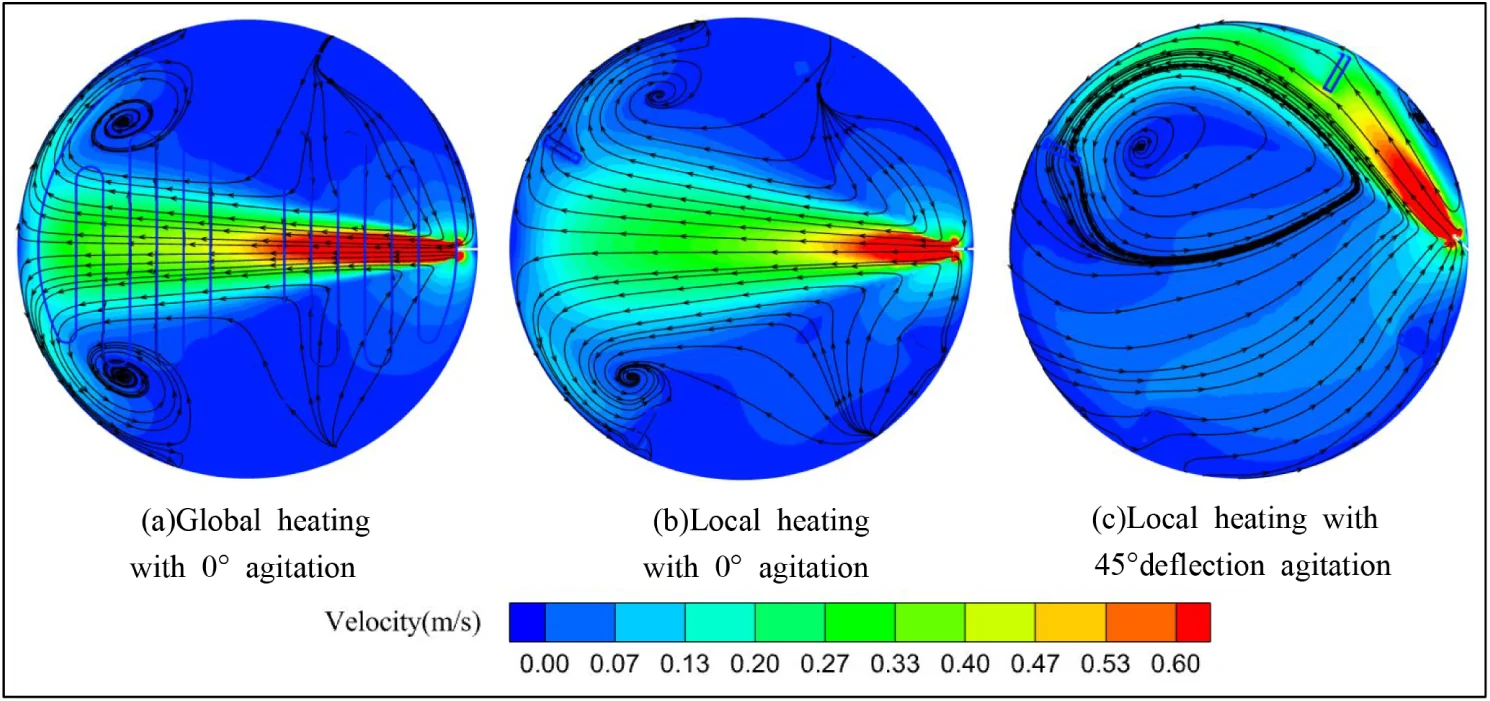
Cloud map of the velocity field at the center section of the agitator after 8.3 h of heating (0.60 m/s is not the maximum or average velocity within the storage tank).

#### 5.1.4 Heat Transfer Analysis Based on Field Synergy Theory

The Field Synergy Principle (FSP) is a significant theoretical framework in the field of heat transfer, primarily employed for analyzing and improving convective heat transfer processes. The core of this principle lies in the fact that the efficiency of convective heat transfer is not solely dependent on the intensity of the flow field (velocity field) and the temperature field, but also critically hinges on the degree of synergy between them. The field synergy angle serves as a key metric for quantifying such synergy, defined by the following eq 18.

18 $$\cos\beta =\frac{\mathrm{U⃗}\cdot \nabla T}{\left|\mathrm{U⃗}\right|\cdot \left|\nabla T\right|}$$

Since waxy crude oil exhibits non-Newtonian behavior at low temperatures, the flow and heat transfer processes become considerably more complex, leading to intensified coupling between the velocity and temperature fields. In this study, the average field synergy angle inside the tank at the end of the heating process was extracted, with the results presented in Table 5. Under tube-only heating conditions, the average field synergy angles were 101.79° for global heating and 112.06° for local heating, indicating poor flow-thermal coupling and consequently unsatisfactory overall heating performance. With the introduction of mechanical agitation, the flow-thermal coupling was improved: the average field synergy angles were 97.58° for global heating with 0° agitation and 96.72° for local heating with 0° agitation. When local heating was coupled with 45° deflection agitation, the average synergy angle further decreased to 94.64°. This reduction demonstrates that adjusting the agitator deflection angle enhances the synergy between the velocity field and the temperature gradient, enabling the flow field to distribute thermal energy from the heat source more effectively throughout the tank while reducing energy loss during heat transfer. Consequently, this configuration enhances the heating performance. Specifically, when using a 45° deflection agitation method, the overall effect of local heating is superior to that of the other four heating methods.

**Table 5:** Average Field Synergy Angle Within the Tank after 8.3 h Heating under Different Heating Methods

Heating method	Only global heating	Only local heating	Global heating with 0° agitation	Local heating with 0° agitation	Local heating with 45° agitation
Average Field Synergy Angle	101.79°	112.06°	97.58°	96.72°	94.64°

Figure 14 illustrates the regions within the tank where the field synergy angle is below 90°. Under tube-only heating conditions, the proportions of regions with a synergy angle below 90° are 10.9% for global heating and 3.4% for local heating. With the introduction of mechanical agitation, these proportions increase to 15.4% for global heating with 0° agitation and 17.36% for local heating with 0° agitation. When local heating is coupled with 45° deflection agitation, the proportion further rises to 19.41%. This indicates that under the local heating with 45° deflection agitation configuration, the flow direction in a larger portion of the tank tends to be more parallel to the temperature gradient compared with the axial (0°) agitation case, thereby directly facilitating heat transfer rather than relying solely on conduction or counterproductive convection. In conjunction with the variable-viscosity characteristics of non-Newtonian fluids, this increase in positively synergistic regions contributes to mitigating the high-viscosity stagnant zones in low-temperature areas, further improving overall heat-transfer efficiency.

**Figure 14:**
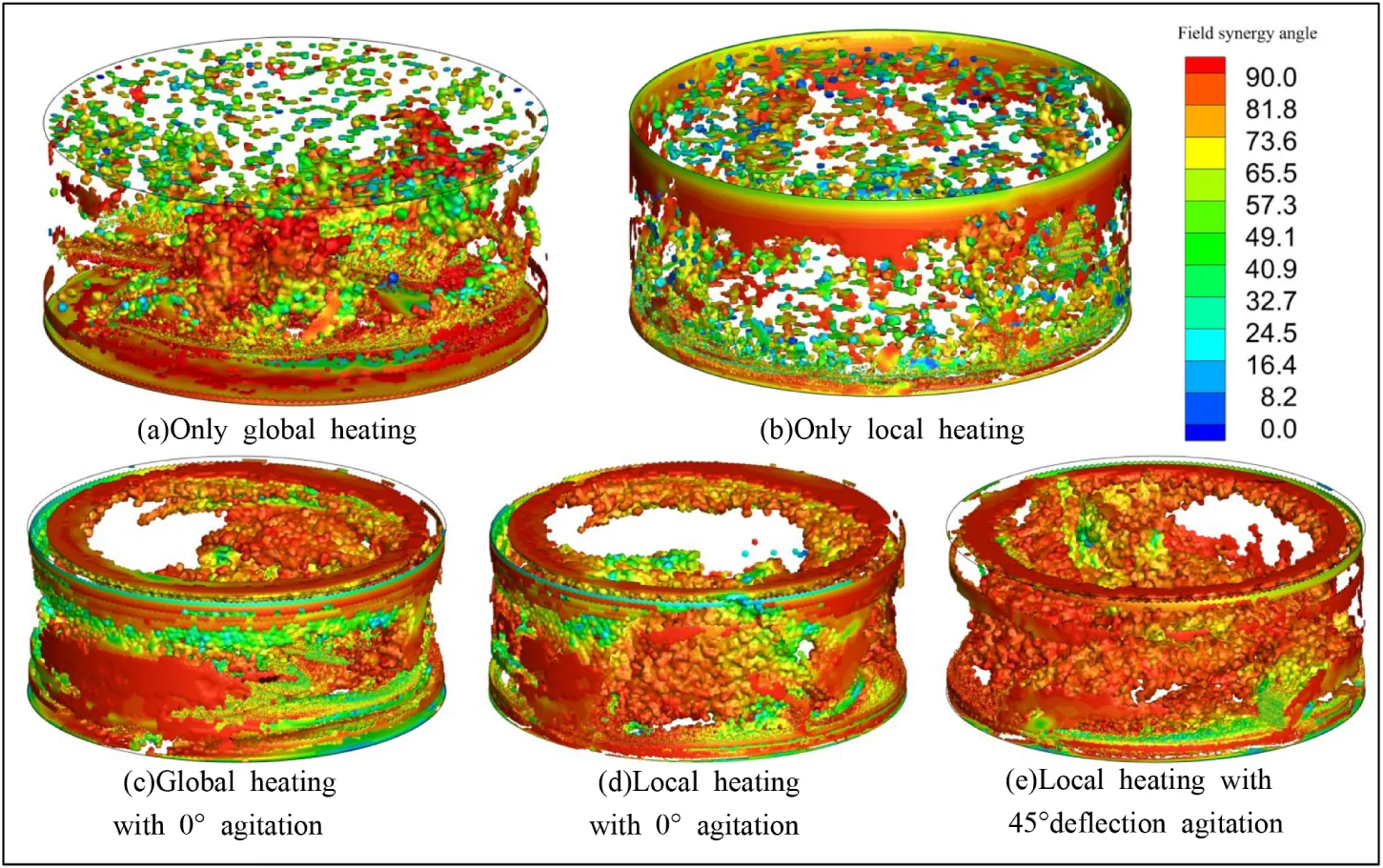
Regions with internal synergy angle below 90° in the tank.

#### 5.1.5 Gel Crude Oil Conversion Effect

At low temperatures, waxy crude oil precipitates the wax crystals. As the temperature decreases, the viscosity of the crude oil increases, leading to a decline in flow capacity and eventually forming a gel-like structure that develops into gelled crude oil. The formation of gelled crude oil not only reduces the effective capacity of storage tanks but more critically, can clog inlet and outlet pipelines, impede the movement of floating roofs, increase restart difficulties, and even cause tank solidification accidents. Consequently, the conversion efficiency of gelled crude oil within the tank also serves as a key indicator for evaluating the heating efficiency of storage tanks.

On the basis of the initial gelled crude oil volume of 75.30 m^3^ (volumetric proportion: 13.32%), the conversion effects of different heating methods were systematically analyzed by examining changes in gelled crude oil volume, distribution cloud maps of gelled crude oil, viscosity variations, and quantitative conversion rate data. As shown in Figure 15, when tubular heating was applied alone—whether global or local heating—the conversion of gelled crude oil in the tank was very limited. At the end of the heating process, global heating and local heating converted only 33.54% and 25.5% of the gelled crude oil, respectively. This indicates that tubular heating alone had a very limited effect on reducing gelation of the crude oil region. After heating, the volumes of gelled crude oil at the tank bottom, wall, and roof remain almost unchanged. Combined with the temperature field analysis presented earlier, it can be seen that, under local tubular heating, the gelled crude oil at the tank roof even increases. Relatively speaking, global heating was slightly better than local heating, but the difference between the two was small, suggesting that, under local tubular heating, the temperature rise in the low-temperature dead zones inside the tank was very limited and insufficient to promote the conversion of the gelled crude oil to a sol state.

**Figure 15:**
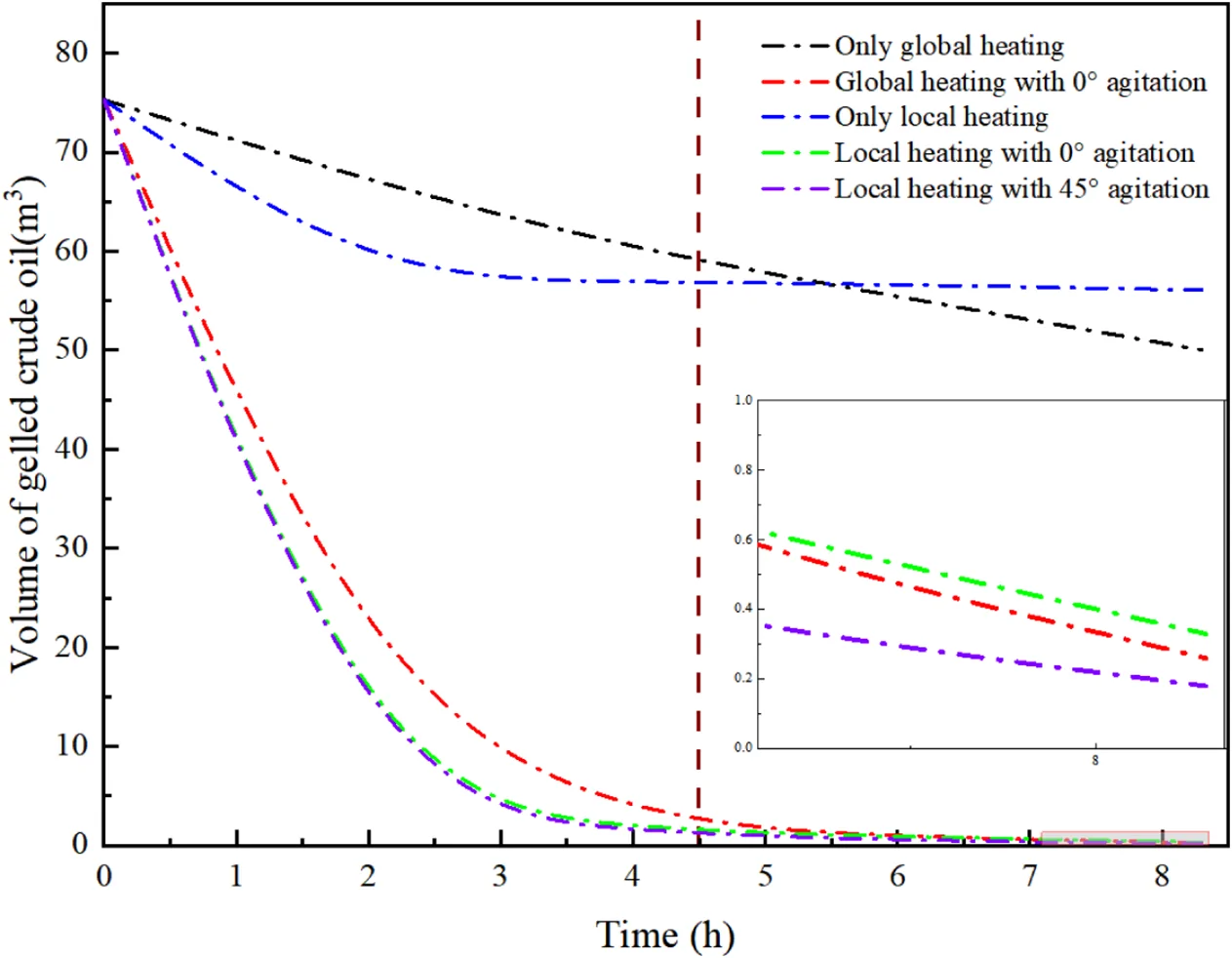
Volume variation of gelled crude oil within the tank during the heating process.

Figure 16 illustrates the initial distribution of gelled oil (Figure 16a) and viscosity within the tank (Figure 16b). At the initial moment, it can be clearly observed from the contour map that the solidified oil is mainly distributed in the tank bottom, along tank wall edges, and on the tank roof (with the largest amount at the tank bottom). Correspondingly, these areas exhibit significantly elevated viscosity levels. These regions also correspond to the low-temperature dead zones and weak-flow regions previously identified.

**Figure 16:**
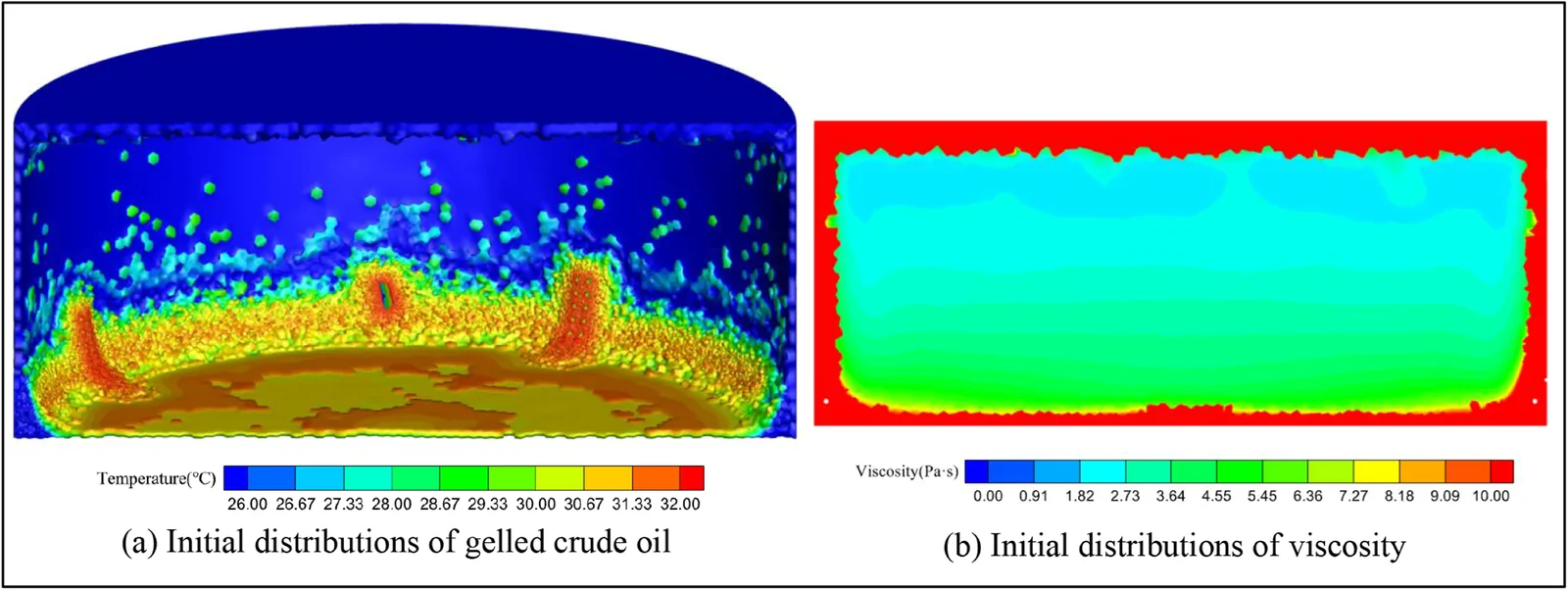
Cloud maps of initial distributions of gelled crude oil and viscosity in the tank.

Following the introduction of mechanical agitation, the gelled crude oil conversion efficiency was substantially enhanced (Figure 17). After 2 h of heating, the gelled oil volume notably decreased from the initial state; local heating with 45° deflection agitation (Figure 17c) demonstrated the most pronounced elimination effect, with only a small area behind the agitator (red dashed box) remaining gelled, whereas melting under global heating with 0° agitation (Figure 17a) and local heating with 0° agitation (Figure 17b) was considerably limited. By 4 h, the gelled oil had further decreased, and the bottom gelled oil under global heating was also largely melted. At 6 h, the gelled oil under both local heating with 45° deflection and local heating with 0° agitation in the lower wall region had mostly melted, while that under global heating with 0° agitation remained largely un-melted, with residual gelled oil persisting in the axial region away from the agitator at the tank bottom (black dashed box). After 8.3 h, the gelled oil volumes under all three agitation conditions were significantly reduced compared to the no-agitation case, with conversion rates all exceeding 99%. The impinging flow generated by the agitator disrupted the flow field, providing a macroscopic driving force for heat transfer, which effectively minimized low-temperature dead zones and expanded the wax crystal melting range. At the final time point, the volume of gelled crude oil in the tank under local heating with 45°-deflected agitation was only 0.18 m^3^ (the conversion rate reached 99.75%), accounting for 0.032% of the total volume (compared to 0.26 m^3^ and 0.33 m^3^ for global heating with 0° agitation and local heating with 0° agitation, corresponding to 0.046% and 0.058%, respectively). The gelled crude oil distribution cloud maps also reveal that the tank wall near the bottom and the bottom region in the local heating with 45°-deflected agitation setup were almost free of gelled crude oil, whereas global heating with 0° agitation and local heating with 0° agitation still exhibited minor residual gelled crude oil in these areas, indicating that the low-temperature dead zones at the tank bottom were substantially eliminated.

**Figure 17:**
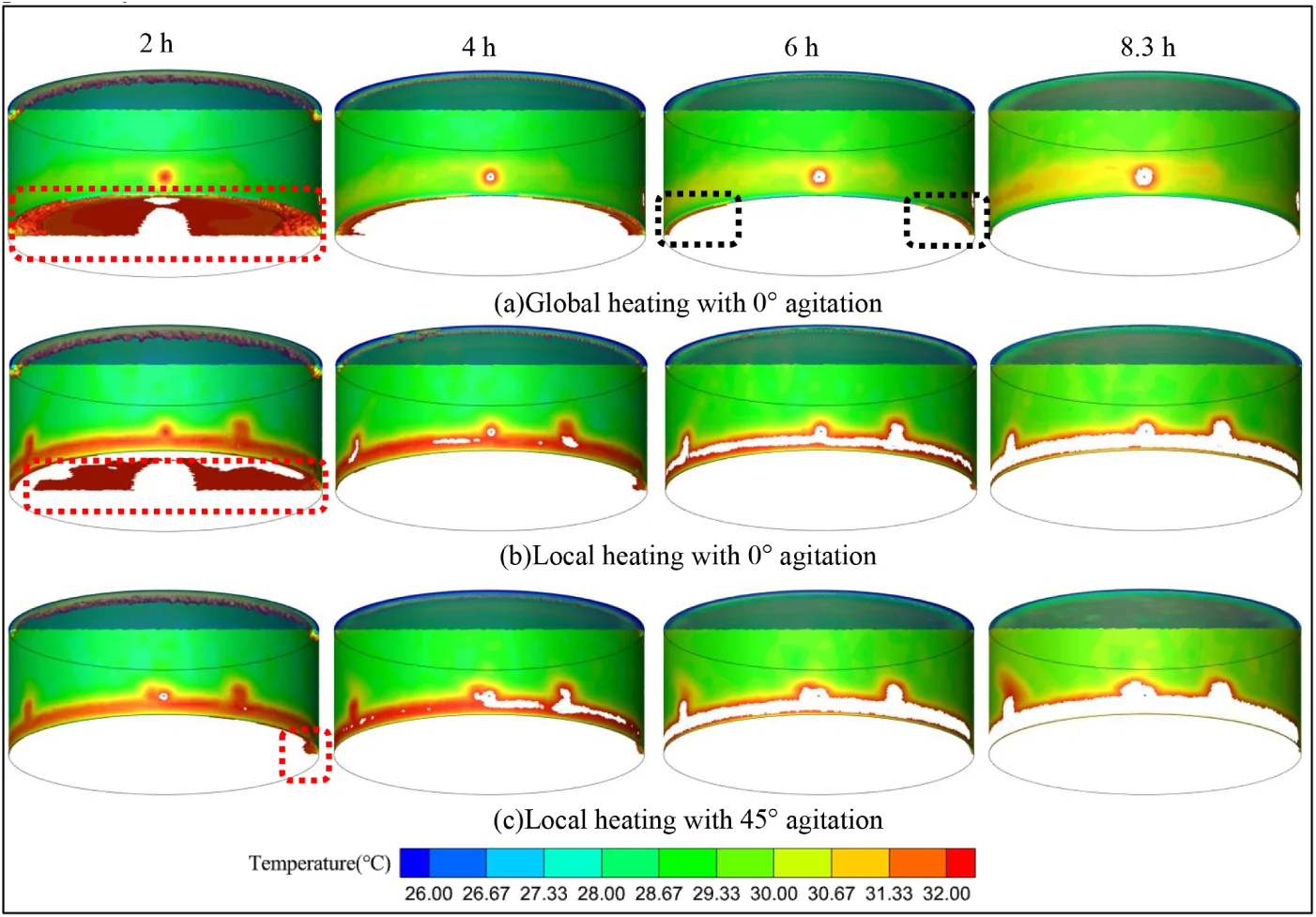
Distribution of gelled crude oil in the tank during heating under different heating methods.

Moreover, the gelled crude oil volume reduction rate under local heating with 45° deflection agitation remained the highest throughout the process. By the 4-hour mark, the gelled crude oil was nearly completely eliminated, underscoring the high conversion efficiency of this heating mode. Although the overall volume reduction differences among the three heating modes were relatively small at the final stage, local heating with 45° deflection agitation demonstrated faster conversion kinetics, higher efficiency, and more thorough elimination. Considering the larger volumes of actual storage tanks, the amount of gelled crude oil converted under this scheme would be greater for the same heating duration, resulting in significant energy savings over extended operations.

Waxy crude oil, under low-temperature conditions, forms a complex three-dimensional network structure due to wax crystal precipitation, exhibiting non-Newtonian fluid behavior with significant shear-thinning characteristics—i.e., its apparent viscosity decreases as the shear rate increases.^41,42^ Therefore, the viscosity of the crude oil in the tank also reflects the intensity of the shear field within the tank. During the cooling process, natural convection predominates in the tank, resulting in generally poor fluidity. By the end of the cooling stage, the average temperature inside the tank approaches the gel point, leading to an overall high crude oil viscosity, as observed in Figure 16b. Particularly in the tank’s wall regions, the crude oil has gelled, exhibiting extremely high viscosity. When mechanical agitation is combined with tubular heating, the crude oil viscosity decreases due to both the temperature increase and the shear-induced reduction in apparent viscosity. As shown in Figure 18, after only two hours of combined mechanical agitation and tubular heating, the viscosity of the crude oil in the tank is significantly reduced. This is especially pronounced under local heating combined with 45° deflected mechanical agitation (Figure 18c): the viscosity of the crude oil near the agitator in the bottom region (within the blue dashed box in Figure 18) drops below 1.0 Pa·s, while only a small portion of high-viscosity oil remains in areas farther from the agitator. In contrast, under global heating with 0° agitation (Figure 18a) and local heating with 0° agitation (Figure 18b), the proportion of high-viscosity crude oil in the same bottom region is significantly larger.

**Figure 18:**
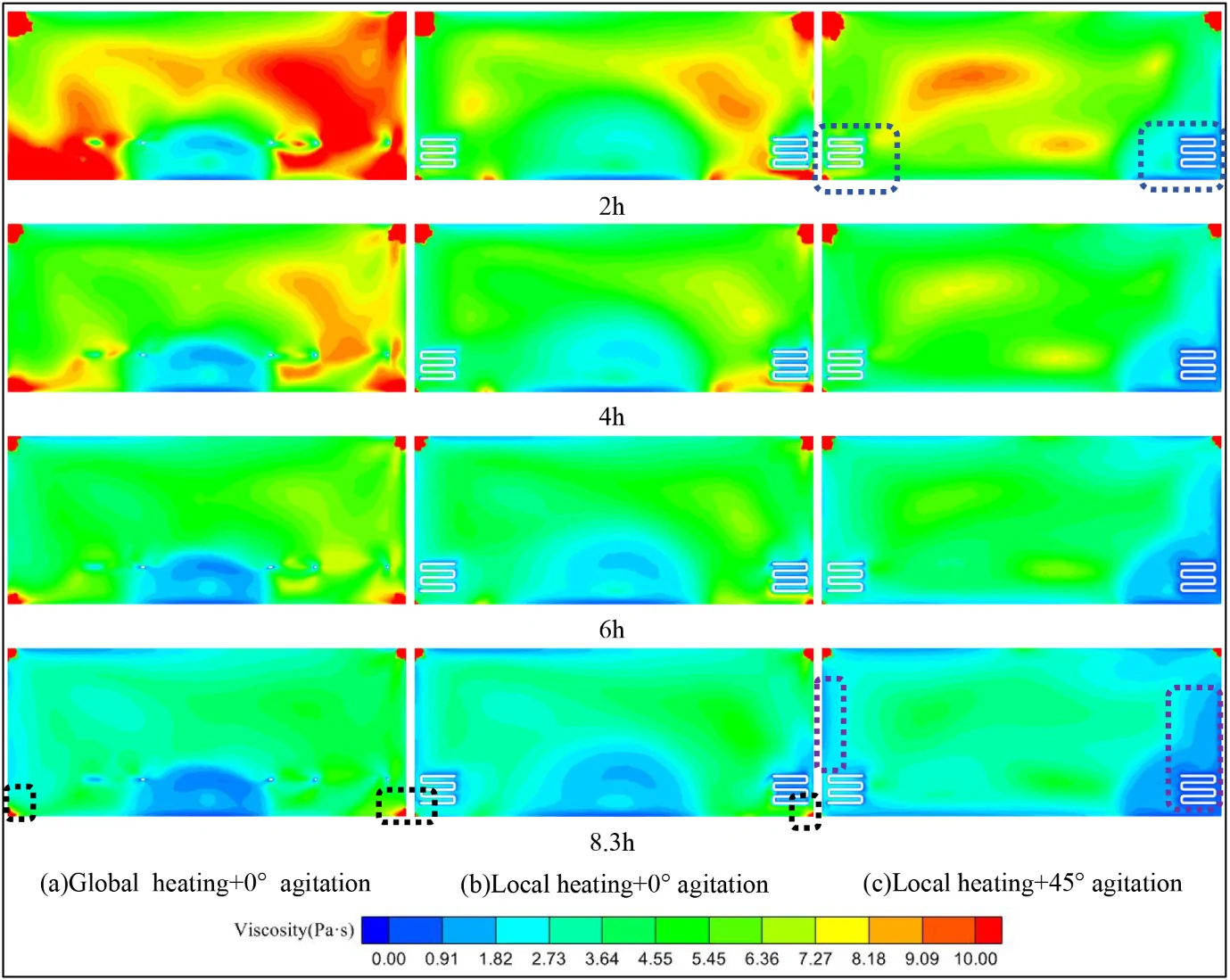
Viscosity variations of crude oil in the tank during heating processes with different methods.

The flow field generated by 0° axial agitation is predominantly characterized by axial circulation with limited radial and circumferential mixing, resulting in insufficient flow coverage and shear uniformity, delayed heat transfer in peripheral regions, and slower wax crystal dissolution and gel breakdown compared to deflected agitation. Under global heating with 0° agitation, extensive high-viscosity areas persist, exhibiting the poorest viscosity reduction and uniformity due to the diffuse heat input that prevents a concentrated heat source and undermines the synergistic shear-enhancing effect. After 4 h, the viscosity under global heating with 0° agitation decreases, but a large high-viscosity area remains at the tank bottom, whereas under local heating with 45° deflection, high-viscosity crude oil is almost entirely eliminated from the bottom region. By 6 h, viscosity further decreases under all three modes. At 8.3 h, the average viscosity under local heating with 45° deflection is 3.12 Pa·s, compared to 3.89 Pa·s for local heating with 0° agitation and 4.03 Pa·s for global heating with 0° agitation. More notably, under local heating with 45° deflection, the low-viscosity region along the tank wall (purple dashed box) occupies a larger area, and high-viscosity crude oil is virtually absent at the tank bottom, whereas significant high-viscosity regions (black dashed box) persist under both 0° agitation configurations.

The tank base wall serves as the primary deposition zone for precipitated wax crystals, exhibiting extremely high initial viscosity and functioning as the core accumulation region of gelled crude oil. It is also the most difficult area to heat up and transform the gelled crude oil under individual tubular heating conditions. As clearly illustrated in Figure 19a, under the scenario combining local heating with 45° deflection agitation, the viscosity reduction at the tank base wall is characterized by the fastest rate and the greatest magnitude after two hours of heating. This outcome is primarily attributed to the concentrated high-temperature heat source generated by the local heating tubes, while the oblique impact flow induced by the 45° deflection agitation directly scours the tank base wall. This configuration not only facilitates efficient heat transfer to the dead zones at the base wall but also continuously shears and disrupts the deposited wax crystal network, which is consistent with the distribution of gelled crude oil depicted in the cloud maps presented earlier. As heating progresses to 8.3 h, nearly no high-viscosity crude oil residues remain at the tank base wall, with only minor regions of high-viscosity oil observed at the edges distant from the agitator. In contrast, under both local heating with 0° agitation and global heating with 0° agitation conditions, the uniformity and rate of viscosity reduction at the tank base wall are significantly inferior. The flow field generated by 0° axial agitation predominantly follows an axial circulation pattern, resulting in weak scouring effects on the tank base wall. Consequently, high-viscosity zones persistently exist in the corner areas of the tank base wall, leading to a marked delay in viscosity reduction.

**Figure 19:**
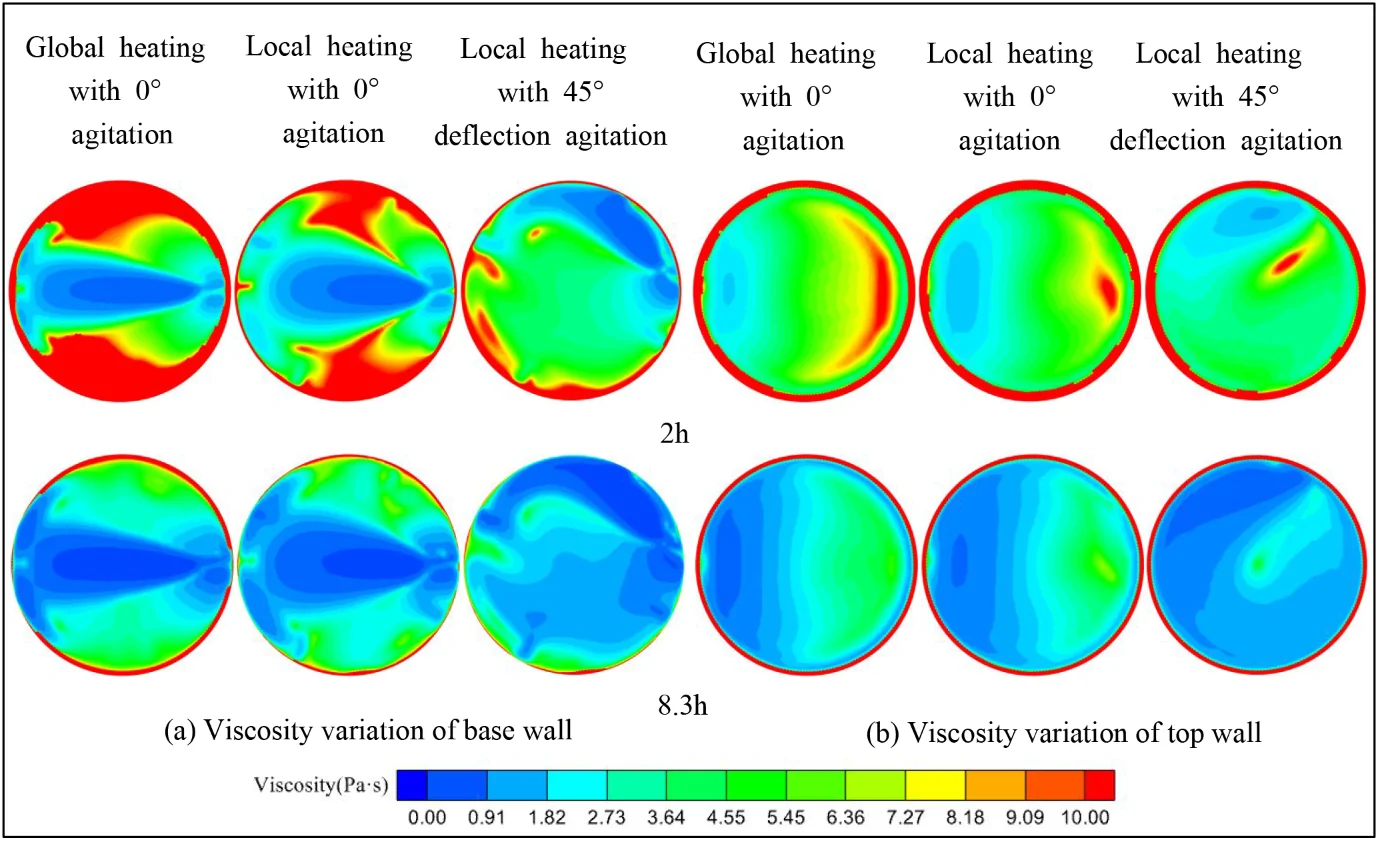
Changes in the viscosity of crude oil of the base wall and top wall under different heating methods.

The tank top wall is another critical low-temperature dead zone within the storage tank. As previously analyzed, under localized heating alone, condensation and accumulation of crude oil on the top wall may even intensify. However, with the introduction of mechanical agitation, the high viscosity in the top wall region is significantly alleviated (Figure 19b), with viscosity decreasing substantially compared to both the initial state and the standalone heating case. This improvement is attributed to forced convection disrupting the thermally stratified structure, continuously circulating high-temperature crude oil from the lower section to the top wall region, while flow disturbance creates effective shear effects that break down the wax crystal gel network. Nevertheless, significant differences remain under various operational conditions. The combined method of local heating with 45° deflection agitation showed the best effect among the five heating methods studied: after 8.3 h, the high-viscosity zone (>2 Pa·s) occupies only 17.9% of the top wall area, compared to 52.1% for localized heating with 0° agitation and 47.3% for global heating with 0° agitation. Furthermore, the viscosity distribution under 45° deflection is relatively uniform across the top wall except near the edges, whereas the 0° configurations result in distinct left–right stratification, indicating that compared to 0° agitation, the 45° deflection induces stronger and more uniform shear effects at the tank top wall.

### 5.2 Experimental Results

To further validate the efficacy of the simulation results, indoor experiments were conducted using the equipment described in Section 4.1, replicating the operating conditions established in the numerical simulations. The tests encompassed only global heating, only local heating, global heating with 0° agitation, local heating with 0° agitation, and localized heating with 45° deflection agitation. To ensure consistency with the initial temperature field in the simulations, a preliminary static cooling phase was implemented prior to the application of various heating methodologies.

#### 5.2.1 Cooling Process

The second group of sensors (tank center) served as the primary focus of analysis, as they are less affected by interference from the tank wall. Axially, the cooling behavior can be divided into three distinct zones (Figure 20a). The central region (measuring points 4, 5, and 6, corresponding to areas d, c, and b within the black frame in Figure 20b) exhibits uniform and overlapping temperature declines, with the slowest cooling rate and the highest final temperature. This is primarily attributed to internal circulation driven by natural convection, which facilitates even heat distribution. The bottom region (measuring points 1, 2, and 3, corresponding to areas g, f, and e within the red frame in Figure 20b) shows a pronounced temperature gradient: temperatures decrease progressively toward the tank bottom, with a cooling rate faster than that in the central region. This behavior stems from both the natural convection characteristics of the cooled oil sinking downward and heat dissipation to the environment through the supporting surface at the bottom. The upper region (measuring point 7, corresponding to area a within the white frame in Figure 20b) is located near the liquid surface and exhibits the fastest cooling rate, though slightly slower than that at the lowermost measuring point.

**Figure 20:**
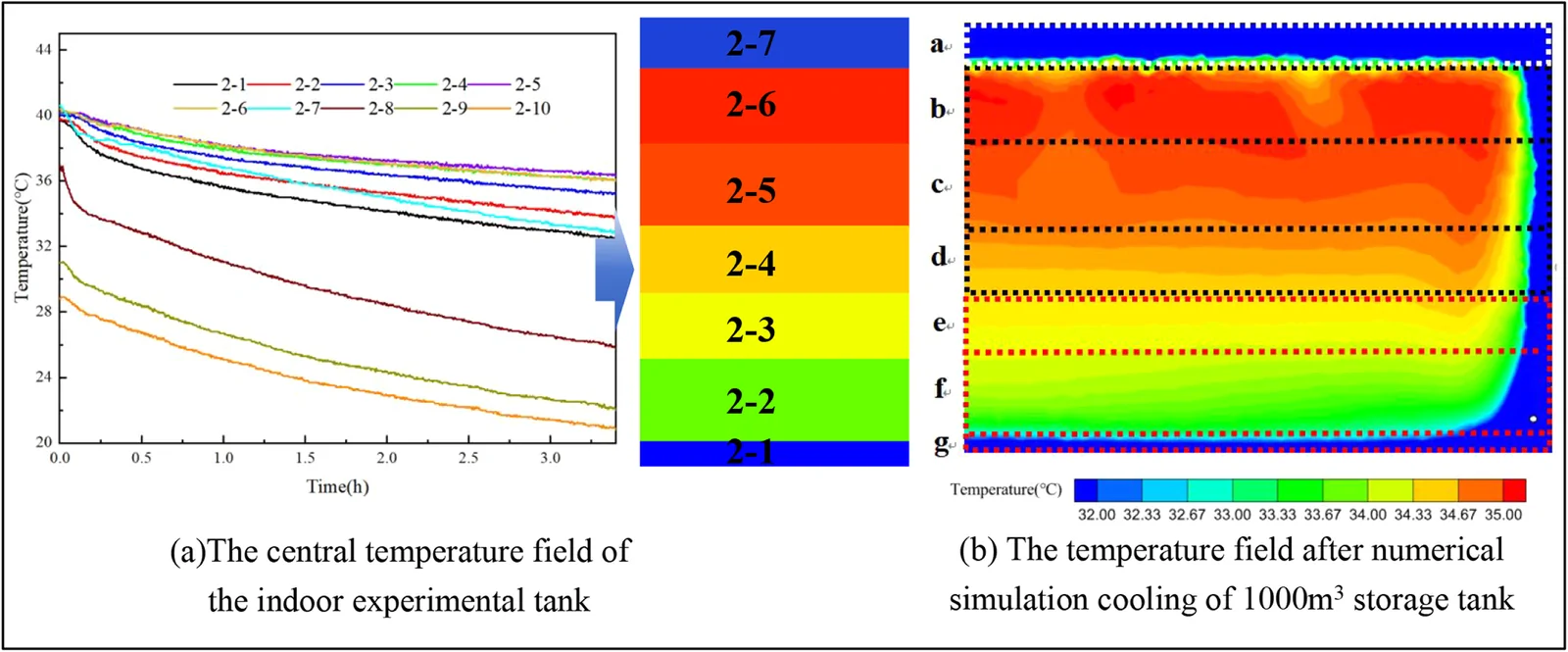
Comparison of experimental and simulation during the cooling process.

In addition, measuring point 8 in the oil vapor region registers a lower initial temperature and cools rapidly, with its variation pattern aligning more closely with gas-phase heat transfer behavior, indicating that this point lies at or above the oil surface rather than within the oil itself. Measuring points 9 and 10 reflect temperature changes in the vapor phase above the oil surface, showing that temperature decreases and cooling accelerates with increasing distance from the liquid surface. The consistent temperatures recorded at measuring points 4–6 in the central region rule out the possibility of pure conductive heat transfer—in the absence of convection, a uniformly increasing temperature gradient would be expected, whereas the presence of temperature-equilibration zones instead confirms natural convection. These experimental observations correspond well with the simulation cloud maps, further validating the accuracy of the numerical modeling approach. The following section presents a comparative analysis of experimental results under different heating conditions.

#### 5.2.2 Heating Process

Initial comparative analysis examined the evolution of average temperatures during the heating process between laboratory-scale experiments (Figure 21a) and 1,000 m^3^ storage tank simulations (Figure 21b), with particular focus on differential temperature responses under various heating-agitation combinations.

**Figure 21:**
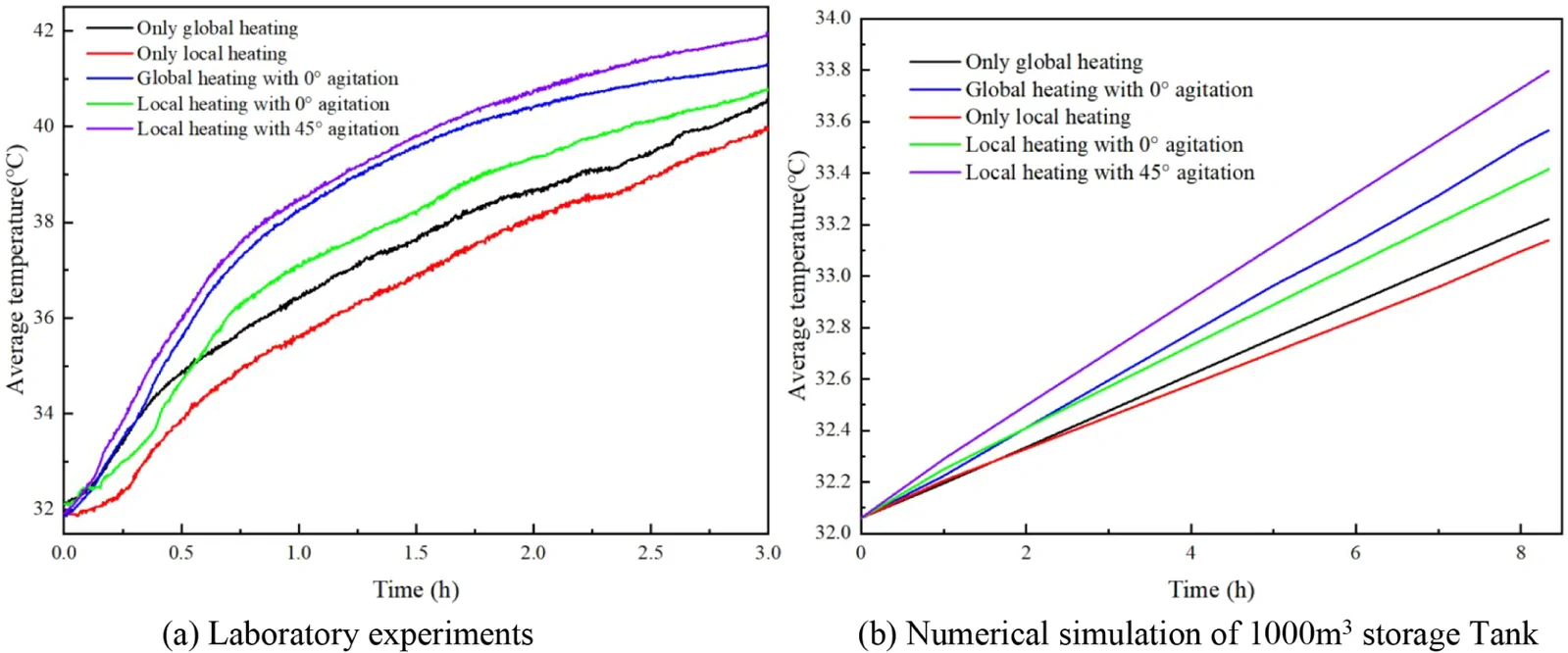
Comparison of experimental and numerical simulation results.

Overall, the experimental and numerical simulation results exhibit generally consistent trends. In the scaled-down indoor tank experiments, among the five heating methods, local heating combined with 45° deflected agitation achieved the highest average heating rate and final temperature, demonstrating better heating performance; this was followed by global heating with 0° agitation, local heating with 0° agitation, only global heating, and only local heating. During the heating process, slight fluctuations were observed in the temperature curves of the indoor experiments, particularly in the initial stage, which is mainly attributable to the arrangement of temperature sensors, introducing a minor discrepancy between the computed average temperature and the actual value. Nevertheless, the overall evolutionary trend aligns well with the numerical simulation outcomes. In the full-scale storage tank simulation, it is evident that local heating combined with 45° deflected agitation yields better heating performance than the other four heating methods. These results also further confirm the validity of the numerical simulation methodology employed in this study.

Figure 22a–e present a comparative analysis of temperature variations at the same location inside the tank under different heating methods in laboratory experiments. Taking the data from positions 3, 4, 5, and 6 of the second sensor group as an example, during only tubular heating, the temperature rise curves at various points reveal a significant thermal gradient within the tank. Under only global heating, the heating rate progressively decreases from bottom to top. In contrast, during only local heating, the absence of heating tubes at the center of the tank results in the lowest heating rate at the bottom. The central and upper regions, due to their higher initial temperatures and lower heat dissipation, exhibit faster temperature increases. This indicates a substantial temperature gradient under only local heating. When mechanical agitation is introduced, the temperature differences among various points in the tank are significantly reduced. Among the tested configurations, local heating with 45° agitation yields the best performance, achieving the smallest temperature differential and the highest average heating rate (2.86 °C/h), compared to 2.63 °C/h for global heating with 0° agitation and 2.59 °C/h for local heating with 0° agitation. Figure 22f further illustrates the temperature response near the heating tubes: under tube-only heating, flow is weak and heat transfer is dominated by conduction and natural convection, causing heat accumulation and a rapid temperature rise around the tubes, especially for local heating with a concentrated heat source. When mechanical agitation is introduced, enhanced fluid motion accelerates heat diffusion, slowing the temperature rise near the tubes, though local heating still exhibits a faster initial increase. A comprehensive comparison confirms that local heating with 45° agitation delivers superior heating performance and overall heat transfer efficiency among the tested configurations.

**Figure 22:**
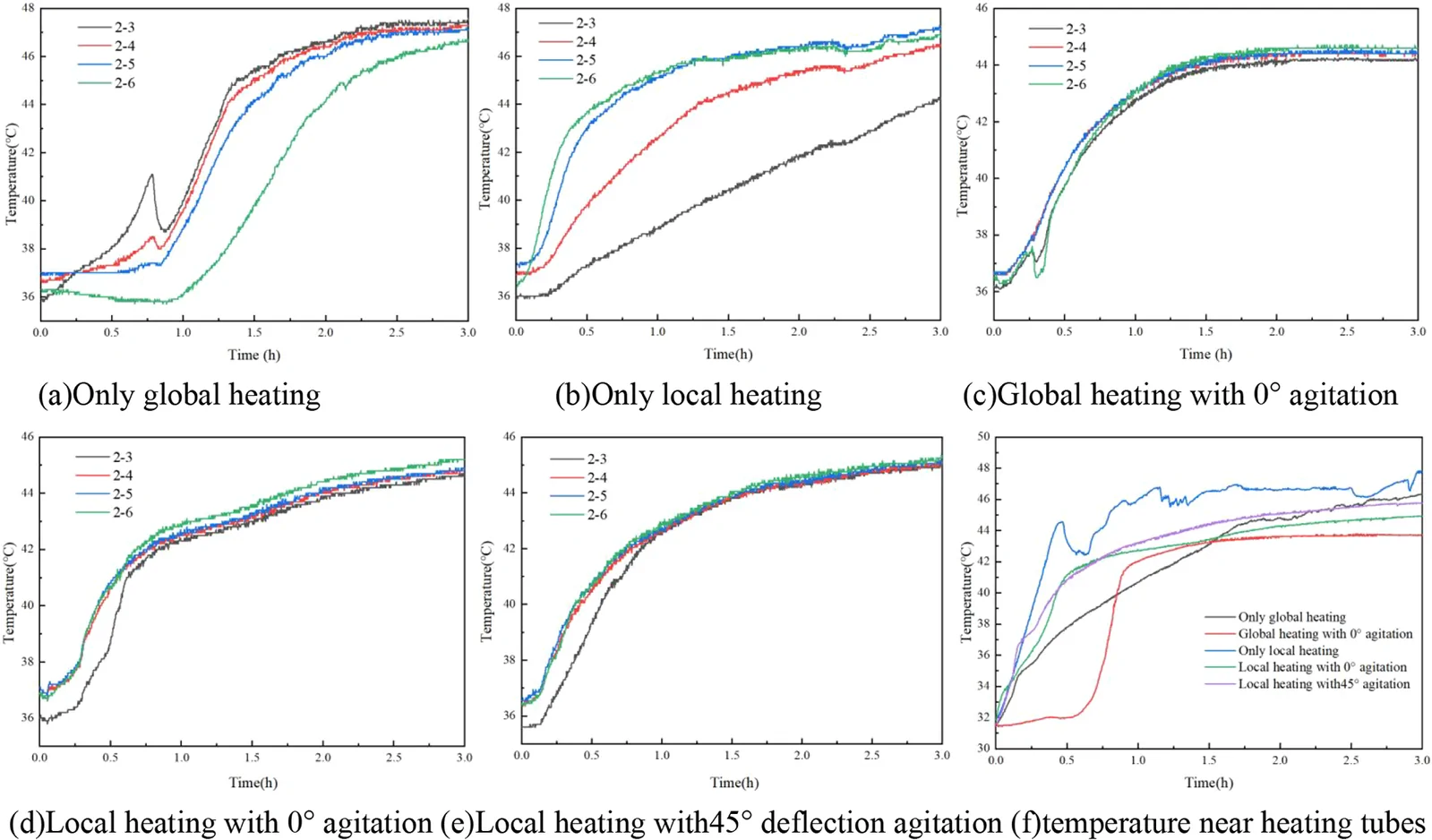
Comparison of the heating process under different heating methods.

Figure 23 compares the melting effects of gelled crude oil on the top of the tank under different heating methods after two hours of heating, with the initial surface condition being gelled as shown in Figure 23a. The white areas in the figure represent the melted crude oil. It is evident that the combined method of local heating with 45° deflection agitation achieves (Figure 23f) the largest melted area after two hours, with only a small portion of gelled crude oil remaining un-melted at the edge away from the agitator. This is followed by global heating with 0° agitation (Figure 23d) and local heating with 0° agitation (Figure 23e), both of which result in similar melted areas, though the residual gelled crude oil area is slightly smaller in the global heating with 0° agitation case. However, the melted areas for both are significantly smaller than that of local heating with 45° agitation. In contrast, only tubular heating (Figure 23b and c) methods only melt a small portion of the gelled crude oil, particularly the only local heating, which only melts a limited area around the central region. These observations align with the conclusions drawn from the numerical simulations presented earlier. The experimental results demonstrate that the combined approach of local heating with 45° deflection agitation yields better heating efficiency and gelled crude oil melting performance than the other four heating methods.

**Figure 23:**
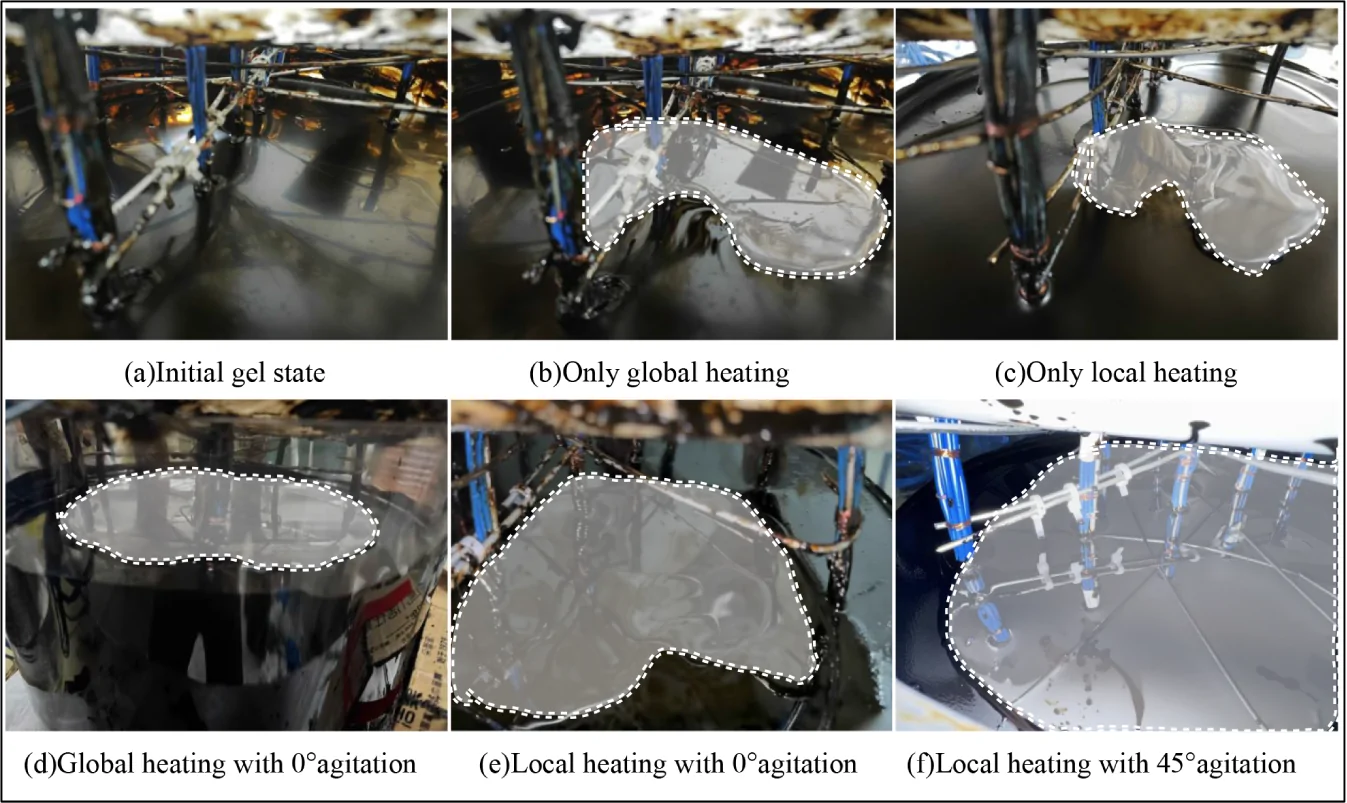
Effects of different heating methods on the melting of gelled crude oil on the top surface of the tank.

#### 5.2.3 Comprehensive Evaluation

To comprehensively assess the heating performance across multiple heating methods, the following evaluation metrics were selected: the average temperature in the tank at the final moment, the average fluid flow velocity, the conversion rate of gelled oil, and the heating efficiency. The calculation of heating efficiency is shown in eq 19. Details are presented in Table 6.

19 $$\eta =\frac{Q_{0}}{Q_{h}}\times 100\%$$

**Table 6:** Various Indicators under Different Heating Methods

Heating methods	Average temperature (°C)	Average velocity (m/s)	Gelled crude oil conversion rate	Heating efficiency
Only global heating	33.22	0.0045	33.54%	56.01%
Only local heating	33.14	0.0023	25.5%	52.33%
Global heating with 0° agitation	33.57	0.047	99.66%	72.07%
Local heating with 0° agitation	33.42	0.059	99.56%	65.19%
Local heating with 45° deflection agitation	33.80	0.068	99.75%	82.63%

*Q*_*0*_ is the energy gained by crude oil after heating, J; *Q*_*h*_ is the heat provided by the heating tube, J.

As evidenced by the data in Table 6, the two heating methods without agitation (only global heating and only local heating) exhibit significant disadvantages. After 8.3 h of heating, the average temperatures inside the tank reached only 33.22 °C and 33.14 °C, respectively, while the average fluid velocities were as low as 0.0045 and 0.0023 m/s. The extremely low flow rates severely hindered effective heat diffusion, resulting in gelled crude oil conversion rates of merely 33.54% and 25.5% and heating efficiencies of only 56.01% and 52.33%, indicating overall low energy utilization efficiency.

With the introduction of mechanical agitation, heating performance improved comprehensively. Under conditions combining global heating with 0° agitation and local heating with 0° agitation, the average flow rates inside the tank increased significantly to 0.047 and 0.059 m/s, respectively. Both configurations achieved relatively high gelled crude oil conversion rates, with heating efficiencies rising to 72.07% and 65.19%, respectively. In contrast, the configuration combining local heating with 45° deflection agitation demonstrated better overall performance than the other four methods. It achieved an average tank temperature of 33.80 °C, an average flow rate of 0.068 m/s, and the highest gelled crude oil conversion rate of 99.75%, along with a heating efficiency of 82.63%. Overall, this heating method outperformed the other four heating methods in terms of heating capacity, flow characteristics, gelled crude oil conversion efficiency, and energy utilization, exhibiting better comprehensive heating performance.

The input energy of the entire heating system comprises three components: the thermal energy provided by the heater to raise the temperature of the heating medium, the kinetic energy supplied by the circulating pump to facilitate fluid circulation within the heating tubes, and the mechanical energy required by the motor to drive the agitator. Specifically, the heater is fueled by natural gas, while both the circulating pump and the stirring motor are electrically powered; this aligns with actual energy consumption patterns in oil and gas field production. In accordance with China’s oilfield equipment operational standards, the circulating pump is assumed to operate at an efficiency of 80%, the heater with a thermal efficiency of 85%, and the accompanying motors are selected from widely used models in oilfield operations. Heater energy consumption is calculated based on the principle of energy conservation, integrating the heater’s thermal efficiency to determine the input energy demand.

The total heat input to the system is mainly partitioned into two components: one portion is absorbed and stored by the crude oil (including both the sensible heat for temperature rise and the latent heat for melting gelled crude oil), and the other portion is dissipated to the environment through the various boundaries (top wall, side wall, and base wall). The macroscopic energy conservation equation for this storage tank system is defined as shown in eq[Disp-formula eq20].

20 $$E_{input}=E_{oil}+Q_{loss-top}+Q_{loss-side}+Q_{loss-base}$$

where *E*_*input*_ represents the total input heat of the system, J; *E*_*oil*_ represents the heat absorbed by the crude oil (including the temperature rise of the crude oil and the latent heat of solidification of the oil), J; *Q*_*loss‑top*_, *Q*_*loss‑side*_, and *Q_loss‑base_,* respectively, represent the heat losses at the top, side, and bottom of the tank, J.

To more clearly illustrate the actual heat dissipation pathways of this heating system, the distribution of input heat is presented in Table 7 as percentages of the total input energy. The results show that under heating without agitation, the energy loss follows the order of side wall > top wall > base wall. When mechanical agitation is introduced, the order shifts to top wall > side wall > base wall. This transition directly reflects the change in the dominant heat-transfer mechanism within the storage tank from natural convection to forced convection. Under tube-only heating conditions, the highly viscous gelled crude oil accumulated at the top wall forms a thick internal thermal insulation layer, causing heat dissipation to concentrate primarily on the side wall, where weak natural convection occurs and the surface area is largest. With the introduction of mechanical agitation, however, the vigorously flowing hot oil is directly transported to the top wall and scours the gelled crude oil, melting and stripping away this insulating gel layer. As a result, the inner surface of the top wall is instantly exposed and brought into direct contact with the high-temperature refluxed crude oil, intensifying the heat exchange. Coupled with the fact that the top wall is directly exposed to an extremely cold external environment, its heat loss rises sharply, eventually making it the largest heat-dissipating surface.

**Table 7:** Percentage of Heat Dissipation and the Average Energy Consumption and Carbon Emissions Corresponding to the Unit Increase in Temperature

		E_loss_(%)				
Heating methods	E_oil_(%)	Top wall	Side wall	Base wall	Average energy consumption per unit temperature rise(kJ)	Average carbon emissions per unit of temperature rise(kgCO_2_/°C)
Only global heating	89.94%	4.61%	5.18%	0.27%	2.589 × 10^6^	259.94
Only local heating	93.32%	2.20%	4.13%	0.35%	2.770 × 10^6^	264.72
Global heating with 0° agitation	86.55%	7.02%	6.02%	0.42%	2.202 × 10^6^	244.76
Local heating with 0° agitation	86.45%	6.99%	6.14%	0.42%	2.435 × 10^6^	250.35
Local heating with 45° deflection agitation	86.34%	7.04%	6.19%	0.42%	1.921 × 10^6^	238.00

As confirmed in our previous analysis, local heating with a 45° agitation configuration substantially enhances the overall heat transfer rate. To achieve the same volume of melted gelled crude oil, the total heating time required is considerably shorter than that under pure heating conditions. Therefore, although its relative heat loss percentage is slightly higher, its average energy consumption per unit temperature rise over the entire operating cycle remains the lowest among all schemes. We have also detailed in Table 7 the average energy consumption and average carbon emissions per unit temperature rise. The results indicate that local heating with 45° deflection agitation achieves the lowest values for both metrics, at 1.921 × 10^6^ kJ and 238.00 kg CO_2_/°C, respectively. Compared to traditional global heating with 0° agitation, these values represent reductions of 12.78% in energy consumption and 2.76% in carbon emissions per unit temperature rise, demonstrating significant potential for energy conservation and emission reduction. This configuration meets the criteria for environmentally friendly upgrades in tank heating systems.

## 6 Conclusion

To address the challenge of gelled crude oil elimination in waxy crude oil storage tanks, this study proposes an improved heating method that couples a local heating tube with an agitator deflection angle (selected as 45° in this work). Through a combined approach of numerical simulation and laboratory experiments, the flow, heat transfer characteristics, and gelled oil elimination performance under different heating-agitation combinations were systematically investigated. Based on the comparative study of five heating methods, the following main conclusions are drawn:

1 The proposed local heating scheme, coupled with 45° deflected agitation, enables near-complete sol-state conversion of the gelled crude oil within the tank in 4 h, achieving a final conversion rate of 99.75%. This is attributed to the rational spatial matching between the flow path and the heating source. The inclined impinging flow generated by the deflected agitator can directly impact the concentrated heating zone, establishing efficient forced convection channels that directionally transport thermal energy to low-temperature dead zones such as the tank base wall and top wall. Meanwhile, the oblique impinging flow exerts mechanical scouring and stripping effects on the gelled oil adhering to the tank walls, directly disrupting the wax crystal network structure of the gelled crude oil and breaking through the thermal resistance barrier formed by the extremely low thermal conductivity of the gel layer, thereby enhancing both overall heating efficiency and gelled crude oil conversion performance of this coupled scheme.
2 Field synergy analysis reveals that local heating coupled with 45° deflected agitation increases the proportion of regions with a synergy angle below 90° to 19.41%, and the improved synergy between the velocity field and the temperature gradient constitutes the fundamental theoretical basis for the enhanced gelled crude oil conversion efficiency. By adjusting the agitator deflection angle to regulate the main flow direction, the effective heat transfer regions where the velocity vector aligns with the temperature gradient are expanded, reducing ineffective energy dissipation during the heat transfer process. Particularly in gel-enriched regions such as the tank bottom and top wall, the enhanced convective heat transfer intensity accelerates the conversion of gelled crude oil, providing a clear theoretical foundation for the high-efficiency gelled crude oil conversion performance of the proposed coupled heating scheme.
3 This coupled process further reduces the energy consumption per unit temperature rise by 12.78% compared to the traditional global heating with 0° agitation scheme, demonstrating the potential to replace conventional global tube heating methods. In long-term daily storage and transportation operations, it can also effectively control gelled crude oil accumulation and reduce tank cleaning and maintenance costs, thereby providing theoretical guidance for the efficient and safe operation and green, energy-saving retrofitting of high-pour-point crude oil storage tanks.

Although this study reveals the coupling mechanism of the improved local heating combined with 45° deflected agitation and its enhancement on heating and gelled oil conversion, our findings cannot prove that 45° is the optimal deflection angle for local heating tubes. Future work will conduct systematic numerical simulations and experiments to refine the optimization of the agitation deflection angle, establish the relationship between deflection angle and heating performance, and thus determine a more precise optimal angle range for the given tank and tube layout. Moreover, in large floating-roof tanks, a single agitator cannot ensure uniform circumferential wall coverage. Future efforts will also focus on the coupled design of multiple heat sources and agitator arrays in ultra-large tanks, investigating flow interference and kinetic energy superposition to achieve uniform circumferential energy distribution and better gelled oil elimination at larger scales, thus promoting industrial application of this novel energy-saving coupling process.

## Data Availability

The data that has been used is confidential.

## Abbreviations

Side wall: Side wall of the storage tank
Base wall: Base wall of the storage tank
Top wall: Top wall of the storage tank
μ: Dynamic viscosity, Pa·s
λ: Coefficient of thermal conductivity, W/m·°C
γ: Shear rate, s^–1^
*T*_*oil*_: Crude oil temperature, °C
C_p_: Specific heat of crude oil, J/kg·°C
*ρ*: Oil density, kg/m^3^
*ρ*_20_: Density of crude oil at 20 °C, kg/m^3^
*β*: Volumetric thermal expansion coefficient of oil
u: Velocity component in *x*-direction, m/s
v: Velocity component in *y*-direction, m/s
w: Velocity component in *z*-direction, m/s
g: Gravitational acceleration, m/s^2^
P: Pressure, Pa
h: Convective heat transfer coefficient, W/m^2^·°C
μ_t_: Turbulent viscosity, m^2^/s
*Nu*: Nusselt number
T_0_: Initial temperature of crude oil, °C
RPM: Revolutions per Minute
